# Piezophilic activity dominates the Puerto Rico Trench deep subseafloor biosphere

**DOI:** 10.64898/2026.09.30.755482

**Published:** 2026-10-01

**Authors:** Miguel Desmarais, Corinna Breusing, Anna H. Finch, Chloé M. J. Baumas, William D. Orsi, Alvaro M. Plominsky, Yuki Morono, Laura M. Wehrmann, Dennis Graham, Robert A. Pockalny, Arthur J. Spivack, David C. Smith, Barbara Saucedo, Sofía Rivera Soto, Jelis Sostre-Cortes, Anne E. Dekas, Jeffrey S. Bowman, Steven D’Hondt, Douglas H. Bartlett

**Affiliations:** 1 Scripps Institution of Oceanography, University of California San Diego, La Jolla, CA, USA; 2 Graduate School of Oceanography, University of Rhode Island, Narragansett, RI, USA; 3 Department of Earth System Science, Stanford University, Stanford, CA, USA; 4 Department of Earth and Environmental Sciences, Ludwig-Maximilians-Universität München, Munich, Germany; 5 Kochi Institute for Core Sample Research, Japan Agency for Marine-Earth Science and Technology, Nankoku, Kochi, Japan; 6 School of Marine and Atmospheric Sciences, Stony Brook University, Stony Brook, NY, USA; 7 University of Puerto Rico at Mayagüez, Mayagüez, Puerto Rico, USA

## Abstract

Hydrostatic pressure is a defining feature of the deep ocean, yet how it structures microbial activity across the subseafloor biosphere is poorly understood. Here, we assess pressure effects on anabolic activity in seafloor and subseafloor communities across a margin-to-hadal-to-abyssal transect of the Puerto Rico Trench region (493–8,385 m water depth). Community-level responses to pressure reverse with burial, revealing a previously unrecognized shift toward piezophily in resident subseafloor communities down to 20 meters below seafloor (mbsf). In pressure-incubation experiments, atmospheric pressure yielded higher proportions of active cells in surficial sediments, whereas *in situ* pressure yielded higher proportions in the subseafloor. Pressure also structured active-community composition; in trench-axis sediments extending to 8.35 mbsf, JS1/Atribacterota populations belonging to geographically widespread hadal lineages exhibited a physiological response to *in situ* pressure, indicative of subseafloor piezophily. These findings suggest that hydrostatic pressure acts as an ecological filter after burial, favoring piezophilic traits that may support microbial persistence in the deep subseafloor biosphere.

The subseafloor biosphere hosts microbial life extending kilometers below the seafloor (Parkes et al. 1994; Whitman et al. 1998; Kallmeyer et al. 2012; Parkes et al. 2014). Although cell abundance and metabolic rates generally decline with sediment depth and age, microorganisms remain metabolically active under conditions of severe energy limitation over geological timescales (Parkes et al. 1994; D’Hondt et al. 2004; Jørgensen and Boetius 2007; Hoehler and Jørgensen 2013). Hydrostatic pressure increases by approximately 1 MPa per 100 m of water depth, reaching 110 MPa (>1,000 atmospheres) in the deepest trenches. It is pervasive across deep-ocean and subseafloor environments and imposes a fundamental constraint on microbial physiology and activity (Bartlett 2002; Lauro and Bartlett 2008; Tamburini et al. 2013; Scoma 2021; Amano et al. 2022). Yet pressure is frequently excluded from experimental studies of the subseafloor biosphere owing to the technical challenges of conducting experiments under *in situ* pressure (Garel et al. 2019).

Pressure has been shown to structure microbial community composition and diverse biogeochemical processes in deep-ocean environments, including organic matter degradation and carbon, nitrogen, and sulfur cycling (Cahet et al. 1990; Bowles et al. 2011; Picard and Ferdelman 2012; Vossmeyer et al. 2012; Garel et al. 2019; Noirungsee et al. 2020; Amano et al. 2022; Stief et al. 2023; Yang et al. 2024; Balmonte et al. 2025; Lloyd et al. 2025; Xiao et al. 2025; Plominsky et al. 2026). In surficial deep-sea sediments, community-level responses to *in situ* pressure range from piezosensitive responses, characterized by lower microbial activity under elevated than atmospheric pressure, to piezophilic responses, characterized by higher microbial activity under elevated than atmospheric pressure (Tamburini et al. 2013). Early experiments with surficial Puerto Rico Trench-axis sediments revealed strongly piezosensitive responses, with bulk heterotrophic activity suppressed under *in situ* pressure, whereas abyssal Atlantic sediments exhibited responses ranging from pressure tolerance to piezophily, with activity maintained or enhanced under *in situ* pressure (Schwarz and Colwell 1975; Tabor et al. 1982; Deming and Colwell 1985). A small number of piezophilic microorganisms have been isolated from deep subseafloor sediments, including sediments buried hundreds of meters to kilometers below the seafloor (Parkes et al. 1995; Bale et al. 1997; Fang et al. 2017), leaving community-level responses to pressure largely unexplored. Consequently, how pressure influences ecological filtering after burial, and the extent to which piezophily contributes to microbial persistence over geological timescales in the deep subseafloor biosphere, remain poorly understood.

Hadal trenches provide a natural setting in which to address this knowledge gap. Their steep bathymetry creates pronounced hydrostatic-pressure gradients across relatively short geographic distances, while topographic focusing and episodic mass wasting generate heterogeneous depositional environments and sedimentary records (Oguri et al. 2013; Turnewitsch et al. 2014; Kioka et al. 2019; Glud et al. 2021; Oguri et al. 2022; Zhao et al. 2026). Where trenches occur near continental margins, these gradients can extend from shallow margin to hadal environments, providing a natural framework for examining microbial pressure responses across hydrostatic regimes and sediment burial depths.

Here, we experimentally assessed community-level responses to pressure across sediment depths ranging from the seafloor surface to 20 meters below the seafloor (mbsf) along a margin-to-hadal-to-abyssal transect in the Puerto Rico Trench region (493–8,385 m water depth). Bioorthogonal non-canonical amino acid tagging (BONCAT) was used to compare the proportion of anabolically active cells in natural communities incubated without added growth substrates under atmospheric and *in situ* pressure; stable-isotope tracing and nanoscale secondary ion mass spectrometry (NanoSIMS) provided complementary single-cell measurements in surficial trench-axis sediments. Fluorescence-activated cell sorting (FACS), metagenomics, and comparative phylogenomics were used to resolve the populations underlying these pressure responses. Together, these approaches reveal how microbial pressure responses change with burial and identify the populations associated with piezophilic activity in the deep subseafloor.

## Results

### Organic matter and redox structure vary across depositional settings

To characterize geochemical gradients associated with microbial habitability across the transect, we examined organic-matter and redox conditions. Total organic carbon (TOC) content was generally highest at margin-proximal PR02, intermediate at PR05, PR06 and PR10, and lowest at PR04, although the greatest values occurred in deeper horizons at the hadal trench-axis sites PR07 and PR08 (Figs. 1c and 2b; Supplementary Table 2). Bulk organic carbon isotopic composition (δ^13^C_org_) was relatively ^13^C-enriched at PR02 and PR05, more ^13^C-depleted at PR04, PR06 and PR10, and most ^13^C-depleted within deeper trench-axis horizons (Extended Data Fig. 1a; Supplementary Table 2).

PR07 and PR08 exhibited an upper carbonate-rich interval and a lower TOC-rich, ^13^C-depleted interval separated at 425 and 575 centimeters below seafloor (cmbsf), respectively (Fig. 1c; Supplementary Table 2). The upper interval was characterized by high calcium carbonate content (CaCO_3_; up to 78.6 wt.%), moderate TOC content (0.18–0.54 wt.%) and comparatively less negative δ^13^C_org_ values (−24.2 to −22.2‰), whereas the lower interval contained uniformly low CaCO_3_ (0.4–1.9 wt.%) and higher TOC (0.77–0.94 wt.%) contents and more ^13^C-depleted organic carbon (−27.7 to −27.1‰), coincident with a transition from lighter- to darker-colored sediment at PR07 (Fig. 2a; Extended Data Fig. 2; Supplementary Table 2).

Across the full dataset, TOC content was strongly negatively correlated with oxygen concentration (ρ = −0.71, *P* < 2.2 × 10^−16^; Fig. 2c; Supplementary Table 4), whereas δ^13^C_org_ showed a weaker negative correlation (ρ = −0.35, *P* = 2.37 × 10^−4^; Supplementary Table 4). Oxygen penetration varied substantially across the transect, with comparatively shallow penetration at PR02, PR05, PR07 and PR08 and deeper penetration at PR04, PR06 and PR10 (Extended Data Fig. 1c; Supplementary Table 3). It broadly decreased with increasing TOC content but differed among intermediate-TOC sites, being shallower at PR05 than at the more ^13^C-depleted PR06 and PR10. Together, these patterns reveal contrasting organic-matter and redox regimes across the transect, with marked vertical geochemical heterogeneity at the trench axis.

### Biomass and diversity covary with organic matter properties

To examine how contrasting organic-matter regimes may support microbial biomass and diversity, we compared biological patterns across the transect in relation to TOC and δ^13^C_org_. Cell abundance, total adenylate (ATP + ADP + AMP), and rarefied amplicon sequence variant (ASV) richness generally remained higher downcore at PR02, PR05, PR07 and PR08 than at PR04, PR06 and PR10 (Fig. 1c; Extended Data Fig. 1e,h; Supplementary Tables 5–7). Across the transect, TOC correlated positively with total adenylate (ρ = 0.48, *P* = 4.97 × 10^−4^) and rarefied ASV richness (ρ = 0.56, *P* = 1.47 × 10^−6^), but its relationship with cell abundance was weaker and not statistically significant (ρ = 0.33, *P* = 0.0584; Fig. 2d–f). δ^13^C_org_ correlated positively with cell abundance (ρ = 0.48, *P* = 0.0106), total adenylate (ρ = 0.54, *P* = 5.06 × 10^−5^) and rarefied ASV richness (ρ = 0.59, *P* = 1.75 × 10^−9^; Extended Data Fig. 3j–l; Supplementary Table 4). Thus, TOC captured broad variation in microbial biomass-linked and diversity proxies, while δ^13^C_org_ also tracked contrasting biological and redox conditions among sites with similar TOC, particularly PR05 relative to PR06 and PR10.

### TOC content decouples from microbial biomass and richness in subseafloor ^13^C-depleted trench sediments

The pronounced heterogeneity in organic-matter content and composition across the trench-axis intervals provided a natural test of their relationships with microbial biomass and diversity. Despite higher TOC content, cell abundance, total adenylate, and ASV richness in the lower interval fell below their transect-wide relationships with TOC (Fig. 2d–f). Mean total adenylate concentration decreased from 9.44 × 10^−4^ to 4.24 × 10^−4^ nM, and mean rarefied ASV richness decreased from 1,571 to 1,432 ASVs (Extended Data Fig. 1e,h; Supplementary Tables 6 and 7). Normalizing total adenylate to cell abundance or biovolume yielded the same pattern, with both metrics decreasing in the lower interval (Extended Data Fig. 1f,g; Supplementary Table 7). Thus, biomass-linked and diversity proxies declined across the transition to the lower TOC-rich, ^13^C-depleted interval, contrasting with their positive relationships with TOC across the broader transect.

### Pressure effects on the proportion of anabolically active cells reverse with burial

To determine how hydrostatic pressure influences the proportion of anabolically active cells with sediment burial across contrasting depositional settings, we used bioorthogonal non-canonical amino acid tagging (BONCAT) in paired atmospheric pressure and *in situ* pressure incubations of surficial and subsurface sediments (Figs. 3 and 4; Supplementary Table 8). Responses yielding higher proportions of anabolically active cells under atmospheric or *in situ* pressure were classified as piezosensitive or piezophilic, respectively, following the activity-based pressure-response framework of Amano et al. (2022). Across all incubations, BONCAT-positive cells comprised 0.4–56.8% of cells.

In surficial sediments, the proportion of BONCAT-positive cells under *in situ* pressure decreased with increasing water depth, from approximately 22% at the shallowest site (PR02; 493 meters below sea level [mbsl]) to <2% at the hadal trench-axis sites PR07 and PR08 (8,359–8,385 mbsl) (ρ = −0.93, *P* = 0.0025, *n* = 7 sites; Fig. 3b). Among the four sites with paired atmospheric and *in situ* pressure treatments, mean proportions of BONCAT-positive cells were 1.2–6.1-fold higher under atmospheric pressure, with the greatest differences at the deeper sites PR10 and PR07. Consistent with this pattern, Pressure Activity Index (PAI) values, calculated as (*in situ* − atmospheric)/(*in situ* + atmospheric), became increasingly negative across the large *in situ* pressure gradient along the transect, reaching −0.72 at PR10 (54 MPa) and −0.70 at PR07 (84 MPa; Fig. 3c).

In contrast, all six paired subsurface horizons yielded mean BONCAT-positive proportions 1.3–3.7-fold higher under *in situ* than atmospheric pressure (Fig. 4a; Supplementary Table 8). This pattern occurred across margin-proximal, bathyal-slope, hadal trench-axis and abyssal-plain sediments (PR02, PR05, PR04, PR07 and PR10). Correspondingly, PAI values were positive across all six subsurface horizons (0.13–0.57), whereas all four paired surficial horizons had negative PAI values (−0.72 to −0.10), demonstrating a consistent reversal in the direction of the pressure response with sediment burial (Fig. 4b).

### Atmospheric pressure enhances single-cell anabolic activity in surficial sediment at trench-axis PR07

To independently assess the piezosensitive response observed by BONCAT in surficial trench-axis sediments, we used nanoscale secondary ion mass spectrometry (NanoSIMS) to measure single-cell ^15^N-ammonium assimilation at PR07 and PR08. Assimilation was quantified as cellular ^15^N atom percent excess (APE) in cells classified as ^15^N-active, as described in Methods. At PR07, the cell-level distribution of ^15^N APE among ^15^N-active cells was higher under atmospheric than *in situ* pressure (adjusted *P* = 4.13 × 10^−5^; Fig. 3f; Supplementary Table 9). At PR08, the cell-level distributions did not differ between pressure treatments (adjusted *P* = 1.00). Nevertheless, atmospheric-pressure incubations at both sites showed broader ^15^N APE distributions and greater cell-to-cell heterogeneity than *in situ*-pressure incubations, with extended upper tails particularly apparent at PR08 (Supplementary Table 9). In contrast, ^13^C-bicarbonate assimilation was negligible across pressure treatments at both sites (Supplementary Table 9). Thus, NanoSIMS provided independent evidence of a piezosensitive response in surficial trench-axis sediment at PR07.

### Active microbial populations differ between pressure regimes in trench-axis sediments

To identify the microbial populations underlying the contrasting pressure responses across sediment depths at trench-axis PR07, BONCAT-positive cells from atmospheric pressure and *in situ* pressure incubations were isolated by fluorescence-activated cell sorting and sequenced (BONCAT-FACS-seq). BONCAT-FACS-seq libraries were generated from 211–3,078 sorted cells per fraction, with genera detected in the sequenced negative control removed before taxonomic interpretation (Supplementary Table 10). BONCAT-positive community composition differed between *in situ* (84 MPa) and atmospheric pressure (0.1 MPa) across sediment depths (Fig. 5e; Extended Data Fig. 4d). In surficial sediment, the *in situ*-pressure BONCAT-positive fraction was dominated by *Omnitrophus* (93.6% of Bracken-estimated genus-level reads), whereas *Pseudomonas* dominated under atmospheric pressure (79.3%), with a smaller contribution from *Psychrobacter*. In subsurface sediments (417–427 and 825–835 cmbsf), *in situ*-pressure BONCAT-positive communities were dominated by JS1/Atribacterota, primarily the Genome Taxonomy Database (GTDB) placeholder taxon 34–128 (90–96%), whereas atmospheric-pressure BONCAT-positive communities were dominated by *Acinetobacter* (82.8% and 99.9%, respectively), with smaller contributions from *Pseudomonas*. Although neither *Pseudomonas* nor *Acinetobacter* was detected in the reagent-only control, both are recognized potential contaminants in low-biomass datasets, and secondary growth during incubation cannot be excluded (Salter et al. 2014; Capo et al. 2021; Fierer et al. 2025); their provenance therefore remains uncertain. In both subsurface horizons, *in situ*-pressure BONCAT-positive communities more closely resembled the corresponding unincubated starting communities (T_0_), determined from sediment preserved immediately after core recovery, than did atmospheric-pressure BONCAT-positive communities, as indicated by Bray–Curtis dissimilarity (Supplementary Table 10). *In situ*-pressure BONCAT-positive communities also showed greater similarity to T_0_ in the subsurface than in the surficial sediment.

To link anabolically active taxa to resident Puerto Rico Trench populations, BONCAT-FACS-seq reads were recruited to the dereplicated T_0_ metagenome-assembled genome (MAG) collection. At both subsurface horizons, *in situ*-pressure BONCAT-positive reads recruited with high confidence to JS1 MAGs within the GTDB 34–128 lineage: PRT_8359_394_Bin36 and PRT_8359_709_Bin1 at 417–427 cmbsf, and PRT_8359_394_Bin36 at 825–835 cmbsf (Supplementary Table 11). Reads from the corresponding below-threshold (B-T) fractions also recruited to these JS1 MAGs, indicating that only a subset of these populations exceeded the BONCAT-positive fluorescence threshold under *in situ* pressure. No surficial BONCAT-FACS-seq reads recruited to the Puerto Rico Trench MAG collection. Thus, distinct populations were active across pressure regimes and sediment depths, with the subsurface *in situ*-pressure response linked specifically to resident JS1/34–128 populations.

### Distinct JS1 populations are enriched in hadal trench-axis sediments

To resolve JS1 distribution and clade structure across the transect, we examined depth-resolved 16S rRNA gene profiles and representative JS1 MAG distributions. Members of the JS1 class within Atribacterota were detected at PR05, PR07 and PR08, with relative abundance and clade composition differing among sites (Fig. 5d; Supplementary Table 12). The three dominant 99%-clustered groups, hereafter termed JS1–16S clades 1–3 to distinguish them from the genome-based clades below, showed clear bathyal–hadal partitioning. At PR05, JS1–16S clade 3 predominated and total JS1 abundance remained low (0–14.11% of 16S reads), whereas trench-axis PR07 and PR08 were dominated by JS1–16S clades 1 and 2, reaching downcore maxima of 77.03% and 76.63%, respectively. This bathyal–hadal pattern was also reflected among the four representative Puerto Rico Trench JS1 MAGs: the two MAGs identified as *in situ*-pressure-active at trench-axis PR07 were present at low relative abundance at PR05 but became increasingly abundant downcore at PR07 and PR08 (Fig. 6c).

JS1 abundance was highest within the lower TOC-rich, ^13^C-depleted intervals at PR07 and PR08 (Fig. 5c,d). Across JS1-containing sites (PR05, PR07 and PR08), JS1 relative abundance increased with TOC (ρ = 0.76, adjusted *P* < 0.001) and sediment depth (ρ = 0.53, adjusted *P* < 0.001), and decreased with oxygen (ρ = −0.77, adjusted *P* < 0.001) and δ^13^C_org_ (ρ = −0.56, adjusted *P* = 0.002; Supplementary Table 13). Thus, distinct JS1 populations were enriched at the hadal trench axis and were most abundant in deeper, organic-rich, ^13^C-depleted and oxygen-depleted sediments.

### In situ-pressure-active JS1 belong to hadal-associated phylogenomic clades

To place the *in situ*-pressure-active JS1 populations in a broader phylogenomic and ecological context, we compared JS1-affiliated genomes from the Puerto Rico Trench and from other marine sedimentary environments. Phylogenomic analysis resolved two hadal-associated JS1 clades comprising Puerto Rico Trench genomes together with genomes from the Atacama, Japan, Kermadec and Mariana trenches (Fig. 6b,e; Supplementary Table 14). These lineages, designated JS1 hadal clades A and B, were nested within the GTDB placeholder taxon 34–128 (phylum Atribacterota, class JS1) and were distinct from non-hadal JS1 genomes recovered from upper-slope and bathyal sediments. Pairwise average amino acid identity (AAI) comparisons placed most JS1 genome pairs, including comparisons between hadal clades A and B, within the genus-like range, whereas species-like values were restricted to subsets within each clade (Extended Data Fig. 6).

The two JS1 MAGs to which reads from *in situ*-pressure BONCAT-positive fractions were recruited belonged to the hadal-associated clades: PRT_8359_709_Bin1 in hadal clade A and PRT_8359_394_Bin36 in hadal clade B (Fig. 6b). In contrast, two representative JS1 MAGs recovered from the shallower PR05 site, PRT_1902_744_Bin5 and PRT_1902_593_Bin9, fell outside the hadal-associated clades and did not recruit reads from *in situ*-pressure BONCAT-positive fractions (Fig. 6b; Supplementary Table 11). Thus, the *in situ*-pressure-active JS1 populations enriched in the lower TOC-rich, ^13^C-depleted interval at PR07 belonged to geographically widespread hadal-associated lineages.

### Hadal-associated JS1 clades are genomically differentiated

To identify genomic features distinguishing hadal-associated JS1 clades from non-hadal lineages, we compared functional profiles, predicted proteome physicochemical properties and genome architecture. KEGG Ortholog (KO)-based functional profiles differentiated JS1 phylogenomic groups, with hadal clade B showing the strongest separation from non-hadal genomes and clade A occupying a region closer to sedimentary organic-matter-associated non-hadal JS1 (Fig. 6d). KO-based functional profiles differed among JS1 genomic groups even after accounting for carbon/substrate regime (PERMANOVA, genomic group R^2^ = 0.156, P = 0.0001), although multivariate dispersion also differed among groups (P = 0.0003; Fig. 6d; Supplementary Table 15).

Clade B had the highest charged-residue fraction, significantly exceeding both clade A and non-hadal genomes, and these differences remained significant after accounting for genome completeness, contamination and genome recovery type (Fig. 6d; Supplementary Tables 16 and 17). Clade B also had a significantly higher KO-assigned ORF fraction than clade A after accounting for these factors (Supplementary Table 17). Thus, hadal-associated JS1 clades were differentiated in functional and predicted proteome properties, with clade B showing the strongest genomic distinction from non-hadal lineages.

## Discussion

Here we report a consistent reversal in microbial responses to hydrostatic pressure with sediment burial in the Puerto Rico Trench region, from predominantly piezosensitive responses in surficial sediments to piezophilic responses in the subsurface. This reversal occurred across contrasting depositional settings, revealing piezophily as a recurring physiological feature of the subseafloor communities examined here. In subsurface hadal trench-axis sediments, dominant JS1/Atribacterota populations belonging to geographically widespread hadal lineages showed a strong response to *in situ* pressure, indicative of piezophily.

In surficial sediment at trench-axis site PR07, a community-level piezosensitive response was supported by both a higher proportion of anabolically active cells and greater ammonium assimilation among ^15^N-active cells under atmospheric relative to *in situ* pressure (Fig. 3b,c,f). Negligible ^13^C-bicarbonate assimilation across pressure treatments was consistent with heterotrophy predominating during the incubation period (Fig. 3f). This pattern mirrors early observations from surficial Puerto Rico Trench-axis sediments, where heterotrophic uptake and cellular assimilation of ^14^C-labeled amino acids were strongly suppressed at *in situ* relative to atmospheric pressure (Schwarz and Colwell 1975). At the community level, such net pressure responses reflect the relative abundance and activity of pressure-sensitive and pressure-adapted populations (Tamburini et al. 2013). Accordingly, continual delivery of particle-associated microorganisms with limited prior pressure exposure may sustain pressure-sensitive populations in surficial sediments across the transect, potentially enhanced by topographic focusing at the trench axis (Turnewitsch et al. 2014; Kioka et al. 2019; Luo et al. 2019; Hiraoka et al. 2020; Stief et al. 2021; Oguri et al. 2022; Chu et al. 2023; Baumas and Bizic 2024; Plominsky et al. 2026). Consistent with this interpretation, the magnitude of surficial PAI increased with water depth, paralleling evidence from deep-water communities that increasing hydrostatic pressure progressively constrains microbial activity, with implications for estimates of carbon cycling at depth (Amano et al. 2022). At PR07, greater cell-to-cell heterogeneity among ^15^N-active cells under atmospheric pressure further suggests that a highly responsive, pressure-sensitive subset may contribute disproportionately to the net pressure response (Fig. 3f; Arandia-Gorostidi et al. 2023).

Below the sediment surface, PAI captured the opposite pattern: *in situ* pressure yielded higher proportions of anabolically active cells across all six subsurface horizons examined (Figs. 4a,b). The consistent shift from piezosensitive surface responses to piezophilic responses after burial suggests that piezophilic physiology becomes advantageous in the subsurface, favoring the persistence of pressure-adapted members of subseafloor communities (Vossmeyer et al. 2012; Tamburini et al. 2013). Over thousands to millions of years, burial under sustained hydrostatic pressure may reinforce this pattern through ecological filtering of surface-derived populations, consistent with the extensive restructuring of microbial communities observed during sediment burial (Jørgensen and Marshall 2016; Starnawski et al. 2017; D’Hondt et al. 2019; Marshall et al. 2019; Zhou et al. 2022). The present experiments cannot resolve whether pressure-adapted populations primarily persisted after burial or proliferated within the subsurface. However, the placement of pressure-active JS1 populations within geographically widespread hadal-associated phylogenomic clades indicates that the observed physiological response is associated with evolutionary differentiation among JS1 lineages. Together with the recurrence of piezophilic responses across all six subsurface horizons and contrasting depositional settings, these observations support a role for sustained hydrostatic pressure as an ecological filter after burial.

Hydrostatic pressure also structured the taxonomic composition of the active fraction. At trench-axis site PR07, *Omnitrophus* dominated the *in situ*-pressure-active fraction in surficial sediment, while JS1/34–128 dominated the *in situ*-pressure-active fractions in both subsurface horizons. Neither was detected in the corresponding atmospheric-pressure-active fractions, consistent with piezophilic physiology in these populations (Fig. 5e; Extended Data Fig. 4d; Supplementary Tables 10 and 11). Comparisons with the unincubated T_0_ communities further supported the surface-to-subsurface reversal in pressure response: active assemblages more closely resembled T_0_ under atmospheric pressure at the surface and under *in situ* pressure in the subsurface (Supplementary Table 10). Together, these patterns show that hydrostatic pressure partitions activity among populations; in the subsurface, piezophilic physiology appears embedded in the resident community rather than confined to a rare subset, consistent with sustained pressure favoring populations capable of functioning *in situ*.

Recruitment of BONCAT-positive reads to resident T_0_ MAGs further linked the subsurface pressure response to JS1/Atribacterota. Within this group, the piezophilic response was lineage-specific: genome-resolved populations active under *in situ* pressure at hadal PR07 were distinct from those characteristic of bathyal PR05. This bathyal–hadal partitioning was also evident among JS1–16S clades, consistent with ecological differentiation along the regional bathymetric gradient (Figs. 5d and 6b,c; Supplementary Tables 11 and 12). The pressure-active trench-axis populations belonged to hadal-associated phylogenomic clades spanning geographically distant trenches, raising the possibility that piezophilic physiology is widespread among hadal JS1 populations (Fig. 6b,e; Supplementary Table 14; Jitsuno et al. 2024; Li et al. 2024; Schauberger et al. 2024). Functional differentiation among JS1 genomic groups, strongest in hadal clade B, persisted after accounting for carbon/substrate regime, raising the possibility that sustained hydrostatic pressure contributes to this divergence alongside other features of the hadal environment (Fig. 6d; Supplementary Table 15). Candidate traits for maintaining cellular function under pressure included an elevated charged-residue fraction in clade B and the genomic potential of Puerto Rico Trench JS1 MAGs to synthesize the compatible solutes trehalose and di-myo-inositol-phosphate, although their contributions to piezophily remain untested (Extended Data Fig. 7; Supplementary Tables 16 and 17; Campanaro et al. 2005; Peoples et al. 2020; Glass et al. 2021; Caliò et al. 2022). Previous studies have shown that JS1/Atribacterota are abundant and transcriptionally active in deeply buried marine sediments, including evidence for populations persisting over geological timescales (Bird et al. 2019; Vuillemin et al. 2020; Mara et al. 2024). Our findings extend observations of JS1 activity to the hadal subsurface and provide evidence of piezophilic physiology, potentially including obligate piezophily, among particular hadal-associated lineages. Adaptation to sustained hydrostatic pressure may therefore contribute to their long-term persistence in the subseafloor biosphere.

The exceptional enrichment of both hadal JS1 clades at the trench axis coincided not only with sustained high pressure but also with a distinctive depositional setting and energetic regime. The trench-axis sedimentary succession consisted of a carbonate-rich upper interval overlying a lower TOC-rich interval in which δ^13^C_org_ reached −27.7‰, the most depleted value among the bulk hadal sediments surveyed in Supplementary Table 18 (Fig. 5c; Extended Data Fig. 1b; Supplementary Table 18; Fryer et al. 2003; Oguri et al. 2013; Luo et al. 2019; Xu et al. 2021; Dutkiewicz and Müller 2022). This contrast may record episodic downslope delivery of margin- or terrestrial-derived organic matter, consistent with a terrestrial organic debrite previously identified in the region (Meyers 1994; Turnewitsch et al. 2014; Kioka et al. 2019; Seibert et al. 2024). Despite its high organic-carbon content, the lower interval supported disproportionately low microbial standing stocks, adenylate content and diversity, potentially reflecting the distinct source and lability of its ^13^C-depleted organic matter together with the low energy yields of anaerobic metabolism (Figs. 1c and 2d–f; Extended Data Fig. 1e–h; Supplementary Tables 5–7; Meyers 1994; Hedges and Keil 1995; Burdige 2007; Arndt et al. 2013; Bradley et al. 2022; Chu et al. 2026). Yet JS1/Atribacterota accounted for up to 77% of 16S rRNA gene reads in this apparent energy-limited interval, among the highest relative abundances reported for marine sediments (Fig. 5d; Supplementary Tables 12 and 13; Inagaki et al. 2003; Newberry et al. 2004; Inagaki et al. 2006; Carr et al. 2015; Lee et al. 2018; Luo et al. 2018; Glass et al. 2021; Han et al. 2023; Jitsuno et al. 2024). Puerto Rico Trench JS1 MAGs encoded pathways for peptide and sugar fermentation and secondary fermentation within bacterial microcompartments, potentially supporting energy conservation and persistence under these anoxic, energy-limited conditions (Nobu et al. 2016; Lee et al. 2018; Bird et al. 2019; Glass et al. 2021; Jitsuno et al. 2024). These patterns suggest that the recurrence and ecological prominence of hadal JS1 lineages across geographically distant trenches may reflect ecological specialization to both sustained hydrostatic pressure and the depositional, redox, and energetic regimes characteristic of trench sediments (Lv et al. 2026).

Together, our findings identify hydrostatic pressure as a key determinant of microbial activity in marine sediments. Its effect reversed with burial, from piezosensitive responses at the sediment surface to piezophilic responses across contrasting subsurface settings, including sediments buried meters beneath the hadal trench axis. This burial-dependent reversal suggests that sustained pressure may act as an ecological filter over long timescales, favoring populations with piezophilic physiology. Because pressure altered both the prevalence and taxonomic distribution of anabolic activity, experiments under *in situ* pressure will be essential for constraining microbial contributions to biogeochemical cycling in marine sediments. More broadly, our findings suggest that piezophilic physiology may be widespread in the deep subseafloor biosphere, providing a new perspective on microbial adaptation and persistence in one of Earth’s largest microbial habitats.

## Methods

### Study area and sample collection

Sediment samples were collected from seven sites (PR02, PR04, PR05, PR06, PR07, PR08 and PR10) spanning margin, slope, hadal trench-axis and adjacent abyssal-plain settings of the Puerto Rico Trench region during cruise AR64–02 (15 February–10 March 2022) aboard the R/V *Neil Armstrong* (Fig. 1a,b; Supplementary Table 1). Sampling sites ranged from 493 to 8,385 meters below sea level (mbsl), corresponding to approximate *in situ* hydrostatic pressures of 5–84 MPa. Sediment cores were recovered using multicore, gravity-core and piston-core systems.

Recovered cores were sectioned into whole rounds on deck and maintained at 4 °C in a shipboard refrigerated van until subsampling. Core temperature was monitored upon recovery to minimize warming during retrieval and handling. Sediment subsamples were collected from the interior of each core section using sterile 60-mL cut-off syringes to minimize contamination from material in contact with the core liner and were subsequently processed or preserved according to downstream analyses.

### Ancillary geochemical, biochemical, and molecular datasets

Geochemical, biochemical, and molecular datasets used in this study included dissolved oxygen (O_2_), nitrate (NO_3_^−^), sulfate (SO_4_^2−^), TOC, total nitrogen (TN), CaCO_3_, δ^13^C_org_, total adenylate (ATP + ADP + AMP), 16S rRNA gene profiles, and sediment metagenomes. O_2_, NO_3_^−^, and SO_4_^2−^ concentrations were measured as previously described by Fischer et al. (2009). Total organic carbon (TOC) content and δ^13^Corg were determined following the method described in Swenson Perger et al. (2023) and Cohen et al. (2024) in the Department of Geosciences at Stony Brook University. TN and CaCO_3_ were measured as described by Trejos-Espeleta et al. (2024). Total adenylate was measured as described by Orsi (2023) and, for samples with paired cell-abundance and biovolume estimates, normalized to cell abundance or total estimated cellular biovolume. Additionally, 16S rRNA gene profiles and the Puerto Rico Trench MAG collection generated from field-collected, unincubated sediment samples were incorporated from Breusing et al. (in preparation); metagenomic assembly, genome binning, quality assessment, and dereplication are described therein.

### 16S rRNA gene analysis and JS1 clade definition

Previously generated 16S rRNA gene profiles from the broader Puerto Rico Trench amplicon dataset were reanalyzed to evaluate depth distributions of JS1-affiliated populations across PR05, PR07 and PR08 (Breusing et al. in preparation). JS1-affiliated ASVs were extracted from the finalized ASV table, and per-sample relative abundances were calculated from read counts normalized to total reads. To evaluate fine-scale JS1 population structure, JS1-affiliated sequence variants were clustered by pairwise sequence similarity using VSEARCH at a 99% identity threshold (Rognes et al. 2016). The three dominant 99%-identity groups are referred to here as JS1–16S clades 1–3 to distinguish them from genome-based hadal clades A and B.

### Sediment cell separation

Microbial cells were separated from sediment using a workflow modified from Morono et al. (2013). The workflow was used for total cell enumeration of unincubated samples and downstream BONCAT and NanoSIMS analyses of incubated samples. Cell separation for total counts was performed at JAMSTEC, whereas BONCAT-associated extractions were performed at the Scripps Institution of Oceanography. Formaldehyde-fixed sediment slurries were subjected to chemical cell detachment using 100 mM EDTA, 100 mM sodium pyrophosphate, 1% (v/v) Tween 80 and methanol, followed by sonication for 20 min in 30-s on/off cycles at 4 °C. Samples processed at JAMSTEC were sonicated in an ice-cooled Bioruptor water bath (Diagenode, Seraing, Belgium) at 20 W, whereas samples processed at Scripps were sonicated on ice using a Model 50 Sonic Dismembrator (Fisher Scientific, Waltham, MA, USA) at 20% power. Cell extracts were separated by density-gradient centrifugation over 30%, 50% and 80% Nycodenz layered above 80% (w/v) sodium polytungstate at 10,000 × *g* for 1 h at 4 °C. The recovered aqueous cell fraction was used for downstream analyses.

### Total cell enumeration

For total cell counts, the recovered cell fraction was diluted 1:100, collected onto 0.2-μm polycarbonate filters, stained with 10× SYBR Green I in phosphate-buffered saline (PBS), and enumerated at JAMSTEC using an automated fluorescence microscopy system based on an Olympus BX-51 microscope (Olympus Corporation, Hachioji, Tokyo, Japan) equipped with an automatic slide handler, LED illumination and a cooled CCD camera, following Morono et al. (2013) and Morono and Inagaki (2010). Mean fluorescent cell diameter was determined from microscopy measurements, and cellular biovolume was estimated assuming spherical geometry (*V* = π*d*^3^/6). Total cellular biovolume was calculated as mean cellular biovolume multiplied by cell abundance.

### Pressure incubations

Before the cruise, artificial seawater (ASW) was prepared from Sea Salts (40 g L^−1^; Sigma-Aldrich, St. Louis, MO, USA; pH ~7), amended with L-cysteine hydrochloride (0.5 g L^−1^) and resazurin (0.5 mg L^−1^) as an oxygen indicator, aliquoted in 30-mL volumes into metalized polyethylene terephthalate pouches (IMPAK Corporation, Sebastian, FL, USA), flushed with ultra-high-purity N_2_ and heat-sealed. The pre-aliquoted ASW was kept cold until sediment processing aboard ship. Pouches were then reopened under continuous N_2_ flushing, and sediment slurry incubations were prepared by combining 30 mL of sediment with 30 mL of ASW (1:1, v/v). Sodium sulfide (150 μL of a 5% [w/v] stock solution; Sigma-Aldrich) was added as a reductant to maintain anoxic conditions. BONCAT incubations were amended with L-homopropargylglycine (HPG; Vector Laboratories, Newark, CA, USA; CCT-1067) to a final concentration of 50 μM, whereas stable-isotope incubations for NanoSIMS were amended with ^13^C-bicarbonate (1.04 mM; Cambridge Isotope Laboratories, Tewksbury, MA, USA; CLM-441) and ^15^N-ammonium (0.65 mM; Cambridge Isotope Laboratories; NLM-467). All shipboard manipulations were performed on ice using pre-chilled ASW, with sediment slurries continuously flushed with ultra-high-purity N_2_ during processing to minimize oxygen exposure. Following addition of sediment and incubation amendments, pouches were homogenized and heat-sealed without headspace. An ASW-only control was included with each pressure-treatment set to assess maintenance of anoxic conditions independently of the sediment incubation controls.

*In situ*-pressure incubations were conducted in custom pin-retained piston-closure pressure vessels filled with distilled water as the hydraulic medium. Sealed incubation pouches were loaded into the vessels and pressurized to *in situ* hydrostatic pressure using an eTensifier electric high-pressure pump system (High Pressure Equipment Company [HiP], Erie, PA, USA). Pressure was measured using the eTensifier integrated pressure transducer. For BONCAT, each sample included two HPG-amended replicates and one HPG-free sediment negative control at *in situ* pressure, with a parallel set maintained at atmospheric pressure. Both atmospheric pressure and *in situ* pressure incubations were maintained at 4 °C for the remainder of the cruise. Pressurized vessels were transported under pressure in a refrigerated van to Scripps Institution of Oceanography, where incubation continued at 4 °C for a total incubation period of 10 weeks to permit detectable HPG incorporation in low-activity deep-sediment communities. Vessel pressure was verified using the eTensifier immediately before depressurization and subsampling at the end of the incubation period. ASW-only controls were opened in an anaerobic chamber and inspected for resazurin color change to verify maintenance of anoxic conditions.

After incubation, pouches were opened in an anaerobic chamber (Coy Laboratory Products, Grass Lake, MI, USA), and matching 5-mL slurry aliquots were collected for downstream analyses. Aliquots for BONCAT and NanoSIMS activity measurements were fixed overnight at 4 °C with formaldehyde at a final concentration of 4%, washed with PBS and 1:1 PBS:ethanol, and preserved in ethanol. Unfixed aliquots for BONCAT-FACS-seq were preserved separately in glycine-TE buffer.

### BONCAT single-cell activity measurements

Extracted cells were prestained with SYBR Green I in solution, collected onto 25-mm, 0.2-μm polycarbonate filters, and washed twice with 3 mL of 1× PBS before on-filter click chemistry following the general framework of Couradeau et al. (2019). The click-reaction mixture contained 100 μM CuSO_4_, 500 μM THPTA, 5 μM Cy5 azide dye (Vector Laboratories, Newark, CA, USA), 5 mM sodium ascorbate, and 5 mM aminoguanidine-HCl in 1× PBS. Filters were incubated with 80 μL of click-reaction mixture for 30 min in the dark under a coverslip. After labeling, filters were washed three times with 3 mL of 1× PBS, cut into quarters, and one quarter was mounted on glass slides in VECTASHIELD mounting medium; the remaining filter material was preserved at −80 °C.

Mounted filter quarters were imaged on a SLIDEVIEW VS200 fluorescence microscope (Olympus Corporation, Hachioji, Japan) using FITC and Cy5 filter sets: FITC/SYBR Green, Ex 474/27 nm, dichroic 495 nm, Em 525/45 nm; and Cy5, Ex 635/18 nm, dichroic 652 nm, Em 680/42 nm. For each replicate filter, a large-area scan covering most of one mounted quarter was acquired and analyzed in QuPath (Bankhead et al. 2017). Total cells were identified from the SYBR Green/FITC channel using the Cell Detection tool, and BONCAT-positive cells were identified from Cy5 fluorescence using a single-image object classifier. Corresponding HPG-free negative controls were carried through labeling and imaging and used to define background Cy5 fluorescence and BONCAT-positive classification thresholds, which were held constant across replicate images within each sample and treatment. Counts of total and BONCAT-positive cells were used to calculate the proportion of anabolically active cells. Pressure effects were summarized using a normalized Pressure Activity Index (PAI), adapted from the pressure-effect metric (Pe) of Tamburini et al. (2013). PAI was calculated from the mean proportion of BONCAT-positive cells across the two incubation replicates for each treatment as (*in situ* − atmospheric)/(*in situ* + atmospheric), with positive and negative values indicating greater activity under *in situ* and atmospheric pressure, respectively.

### NanoSIMS single-cell activity measurements

Fixed cells collected on gold-coated filters were mounted on conductive carbon tabs on glass slides and analyzed using a NanoSIMS 50L (CAMECA, Gennevilliers, France) at the Stanford Nano Facility. Randomly selected areas were pre-sputtered over 40 × 40 μm for 2 min, after which 30 × 30 μm areas were analyzed with a 5-pA Cs^+^ primary ion beam, a 200-μm aperture (D1–3), and a 256 × 256-pixel raster across 40 serial images. An entrance slit of 20 × 140 μm (ES-3) and aperture slit of 80 × 80 μm (AS-4) were used. Secondary ions were collected for ^12^C^−^, ^13^C^−^, ^12^C^12^C^−^, ^12^C^13^C^−^, ^12^C^14^N^−^ and ^12^C^15^N^−^. Mass resolving power was approximately 9,000 as reported by the CAMECA software.

Cellular regions of interest (ROIs) were manually delineated in Look@nanoSIMS (Polerecky et al. 2012) using carbon-, nitrogen- and isotope-ratio images. Between 194 and 377 ROIs were analyzed per sample; ROIs with Poisson error >10% were excluded. Incubations were amended with both ^13^C-bicarbonate and ^15^N-ammonium, and incorporation of both isotopes was evaluated. A T_0_ sample consisting of PR10 sediment fixed immediately after addition of stable-isotope-labeled substrates was used as a common baseline for calculating atom percent excess (APE) and defining thresholds for detectable ^13^C and ^15^N enrichment, with immediate fixation minimizing biological incorporation of the added tracers. Daily measurements were adjusted by one-point correction to a check standard. Cells were classified as ^13^C- or ^15^N-active when their respective atom fraction exceeded the corresponding T_0_ mean by >2 standard deviations, and APE was calculated as described by Pett-Ridge and Weber (2012).

### BONCAT-FACS, genome amplification, and sequencing

To recover DNA from anabolically active cell populations, sediment slurries from three PR07 horizons (0–10, 417–427 and 825–835 cmbsf) were analyzed by BONCAT-FACS-seq. Slurries were incubated with HPG for 10 weeks and subsequently preserved in glycine-TE buffer. Samples were processed using the cell extraction, on-filter DNA staining and BONCAT click-chemistry labeling workflow described above, with Alexa Fluor 405 azide dye (Vector Laboratories, Newark, CA, USA) used as the BONCAT fluorophore and with modifications to preserve cell integrity for sorting. Specifically, the sonication step used during standard cell extraction was omitted, and after labeling and washing, cells were gently resuspended from filters into 1× PBS containing 0.02% Tween 20 for flow cytometric sorting (Plominsky et al. 2026).

Cell populations were sorted on a BD FACSDiscover S8 Cell Sorter (BD Biosciences, Franklin Lakes, NJ, USA) at the Scripps Research Flow Cytometry Core Facility. Instrument performance and optical alignment were verified using the facility’s standard quality-control and calibration procedures before sample acquisition and sorting. SYBR Green fluorescence was excited by the 488-nm blue laser and detected in the B3 channel (532/16-nm bandpass), while red fluorescence was excited by the 640-nm red laser and detected in the R2 channel (675/20-nm bandpass). These fluorescence parameters, together with forward and side scatter, were used to distinguish cellular particles from sediment particles and background events. Within the gated cell population, Alexa Fluor 405 fluorescence (V3 [460]-A) was used to separate HPG-labeled BONCAT-positive cells from the B-T fraction. An HPG-amended copper-omission click-chemistry control prepared from PR02 surficial sediment (0–10 cmbsf; 5 MPa) was used to characterize background Alexa Fluor 405 fluorescence and define the BONCAT-positive sorting threshold. A reagent-only PBS control containing the processing reagents in PBS but no sediment-derived cells was carried through BONCAT-FACS separately, and events within the sort gate were collected for downstream MDA and sequencing to identify reagent- and workflow-associated contaminants. Cells within the parent-cell gate were sorted into a BONCAT-positive fraction and a separate below-threshold fraction comprising cells that did not exceed the Alexa Fluor 405 fluorescence threshold used for BONCAT-positive sorting. The same gates were applied consistently across samples. Sorting was performed in purity mode into low-bind tubes containing PBS. Sample-specific acquired-event counts, sorted-cell counts, sequencing results and read-QC summaries are provided in Supplementary Table 10.

Because sorted-cell biomass was low, cells were lysed and genomic DNA was amplified using the REPLI-g Single Cell Kit (QIAGEN, Hilden, Germany; cat. no. 150343) according to the manufacturer’s single-cell protocol. Following cell lysis and DNA denaturation/neutralization, Phi29 polymerase-based multiple displacement amplification (MDA) was performed at 30 °C for 8 h, followed by polymerase inactivation at 65 °C for 3 min. The reagent-only PBS control was carried through MDA and sequencing alongside the sorted experimental fractions. Accordingly, BONCAT-FACS-seq profiles were interpreted as the taxonomic composition of recovered, detectably labeled cells rather than as quantitative estimates of total-community composition. Amplified DNA was submitted to the DNA Technologies and Expression Analysis Core at the UC Davis Genome Center for Illumina library preparation and sequencing on an Illumina NovaSeq X platform using 2 × 150 bp paired-end sequencing. Sequencing and read-QC summaries are provided in Supplementary Table 10.

### BONCAT-FACS-seq bioinformatic analysis

Raw BONCAT-FACS-seq reads were assessed using FastQC v0.12.1 (Andrews 2010) and adapter- and quality-trimmed using Trimmomatic v0.39 (Bolger et al. 2014) with the parameters ILLUMINACLIP:TruSeq3-PE-2.fa:2:30:10, LEADING:3, TRAILING:3, SLIDINGWINDOW:4:15 and MINLEN:36. Post-trimming read quality was assessed using FastQC and summarized using MultiQC v1.19 (Ewels et al. 2016). Taxonomic classification was performed using Kraken2 v2.14 (Wood et al. 2019) against a custom GTDB release 226 database downsampled to a maximum hash-table size of 128 GB using the Kraken2 --max-db-size option, with a classification confidence threshold of 0.05. Taxonomic abundances were estimated using Bracken v3.1 (Lu et al. 2017) with a read length of 150 bp and a minimum abundance threshold of 10 reads; genus-level profiles were used for downstream community-composition analyses. Genera detected in the sequenced reagent-only PBS control were removed. Relative abundances were recalculated from the retained Bracken-estimated reads after contaminant filtering. Bray–Curtis dissimilarity between each sorted fraction and its corresponding T_0_ metagenomic community was calculated from the resulting genus-level relative-abundance profiles after normalization to unit sum.

For genome-resolved recruitment, quality-controlled paired-end reads were mapped against the Puerto Rico Trench MAG collection using Bowtie2 v2.5.4 (Langmead and Salzberg 2012) with the --very-sensitive-local setting, retaining a single best alignment per read (-k 1) and allowing insert sizes up to 2,000 bp. Primary alignments were processed using SAMtools v1.20 (Danecek et al. 2021), with unmapped, secondary, and supplementary alignments excluded and a minimum mapping quality of 20. MAG recruitment and genome coverage were quantified using CoverM v0.7.0, retaining alignments with ≥95% nucleotide identity across ≥75% of the read. MAG–sample pairs with ≥20% genome breadth of coverage were classified as high-confidence recruitment; remaining non-zero recruitment was considered low-level.

### Phylogenomic analysis

JS1-affiliated MAGs and single amplified genomes (SAGs) from marine sediments were compiled from published studies, associated NCBI genome repositories, and genomes provided directly by principal investigators when available (Supplementary Table 14). Genome quality was assessed with CheckM v1 (Parks et al. 2015), and taxonomy was assigned with GTDB-Tk using GTDB release 226 (Chaumeil et al. 2020) to confirm placement within the JS1 class-level lineage. Genomes were retained for phylogenomic analysis if they had ≥50% completeness and <10% contamination based on CheckM estimates. Genomes yielding all-gap marker alignments were excluded from downstream tree construction. Environmental source metadata for each genome were obtained from the corresponding publication and, where available, associated NCBI metadata. Prior to comparative genomic analysis, sampling environments were manually classified into broader carbon/substrate regimes based on the reported depositional setting and substrate characteristics; genome-specific assignments and source metadata are provided in Supplementary Table 14.

Phylogenomic reconstruction was performed with the non-dereplicated MAG/SAG collection so that closely related genomes were retained and fine-scale lineage structure could be visualized. Conserved bacterial single-copy marker genes were identified and concatenated with GToTree v1.6.31 using the built-in bacterial HMM set in best-hit mode (Lee 2019). Maximum-likelihood phylogenies were inferred from the concatenated protein alignment using IQ-TREE (Nguyen et al. 2015), with ModelFinder used to select the best-fit substitution model (Kalyaanamoorthy et al. 2017) and branch support assessed with 1,000 ultrafast bootstrap replicates (Hoang et al. 2018). The final tree was visualized and annotated in iTOL v7.6 (Letunic and Bork 2021). Pairwise AAI was calculated from predicted protein sets using CompareM v0.1.2 to evaluate genome-relatedness within and between JS1 clades. AAI values of 65–95% and ≥95% were interpreted as approximate genus-like and species-like similarity ranges, respectively, following commonly used genome-relatedness thresholds (Konstantinidis et al. 2017).

### Genome architecture, functional annotation, and physicochemical properties

Genome architecture metrics were calculated for the retained JS1 MAG/SAG set using CheckM v1.2.5 and Prodigal v2.6.3. Outputs included observed genome size, corrected genome size, GC content, predicted ORF count, and predicted ORF density (Hyatt et al. 2010; Parks et al. 2015). Corrected genome size was calculated as observed genome size × 100 / (completeness − contamination), and ORF density was calculated as predicted ORFs per corrected Mbp. Predicted proteins were assigned to KOs using KOfamScan v1.3.0 (Aramaki et al. 2020).

Proteome physicochemical properties were calculated from Prodigal-predicted proteins using the Peptides package (Osorio et al. 2015). Genome-level proteome summaries included protein isoelectric point, acidic:basic residue ratio, charged residue fraction, hydrophobic residue fraction, and amino-acid composition. Genome architecture, KO-assignment, and proteome physicochemical metrics were summarized for non-hadal JS1 genomes and hadal-associated JS1 clades resolved in the phylogenomic analysis.

### Statistical analyses

Statistical analyses were performed in R v4.5.3, with multivariate analyses conducted using vegan v2.7–5 (Oksanen et al. 2001). BONCAT-positive proportions were measured in two incubation replicates per sediment horizon and pressure treatment and were therefore compared descriptively using replicate values, fold differences between treatment means, and PAI. The relationship between water depth and the mean *in situ*-pressure BONCAT-positive proportion in surficial sediments was tested using a two-sided Spearman rank correlation across the seven sites.

Cellular isotope-incorporation analyses were restricted to isotopically enriched (“active”) cells because sediment background prevented reliable identification and enumeration of all unenriched cells in the ^12^C^14^N^−^ and ^12^C^12^C^−^ images. Cellular ^15^N atom percent excess distributions were compared between pressure treatments within each site using two-sided Wilcoxon rank-sum tests with Bonferroni correction for two comparisons. Because each site and pressure treatment comprised one NanoSIMS incubation, these tests compare cell-level distributions within incubations and do not represent incubation-level replication. Gini coefficients were calculated separately from ^13^C and ^15^N APE values of active cells using DescTools v0.99.60 to quantify cell-to-cell heterogeneity in isotope assimilation (Arandia-Gorostidi et al. 2023).

Associations among geochemical variables, biomass-linked proxies, rarefied ASV richness and JS1 relative abundance were tested using two-sided Spearman rank correlations. Variables measured at different sediment depths were paired within sites using maximum-cardinality, minimum-total-offset one-to-one matching without replacement; matching tolerances and median depth offsets are reported in Supplementary Table 4. O_2_ was linearly interpolated to target horizons and set to zero at and below the first measured zero. The 15 transect-wide correlations were adjusted together using the Benjamini–Hochberg procedure. Correlations between JS1 relative abundance and geochemical variables constituted a separate analysis family and were adjusted separately using the Benjamini–Hochberg procedure (Supplementary Table 13).

Group-wise comparisons among non-hadal JS1 genomes and the two hadal-associated clades were performed using pairwise Wilcoxon rank-sum tests, with *P* values adjusted for multiple comparisons using the Benjamini–Hochberg procedure (Supplementary Table 16). These tests were applied to genome architecture metrics, KO-assignment metrics and predicted proteome physicochemical properties. Because genome type and quality differed among JS1 genomic groups (Extended Data Fig. 5), covariate-adjusted linear models were used as robustness checks for selected genome architecture, KO-assignment and proteome physicochemical metrics. These models included JS1 group as the predictor of interest and CheckM-estimated completeness, CheckM-estimated contamination and genome type as covariates. Statistical outputs are reported in Supplementary Table 17.

Multivariate differences in KO-based functional profiles were assessed using Bray–Curtis dissimilarities calculated from KO copy densities normalized per 1,000 predicted proteins. Ordination was performed by principal coordinate analysis (PCoA), and group differences were tested using PERMANOVA with adonis2 in vegan, with additional models including carbon/substrate regime as a covariate. Differences in multivariate dispersion were evaluated using betadisper in vegan. Statistical outputs are reported in Supplementary Table 15.

## Extended Data

**Extended Data Fig. 1 |:**
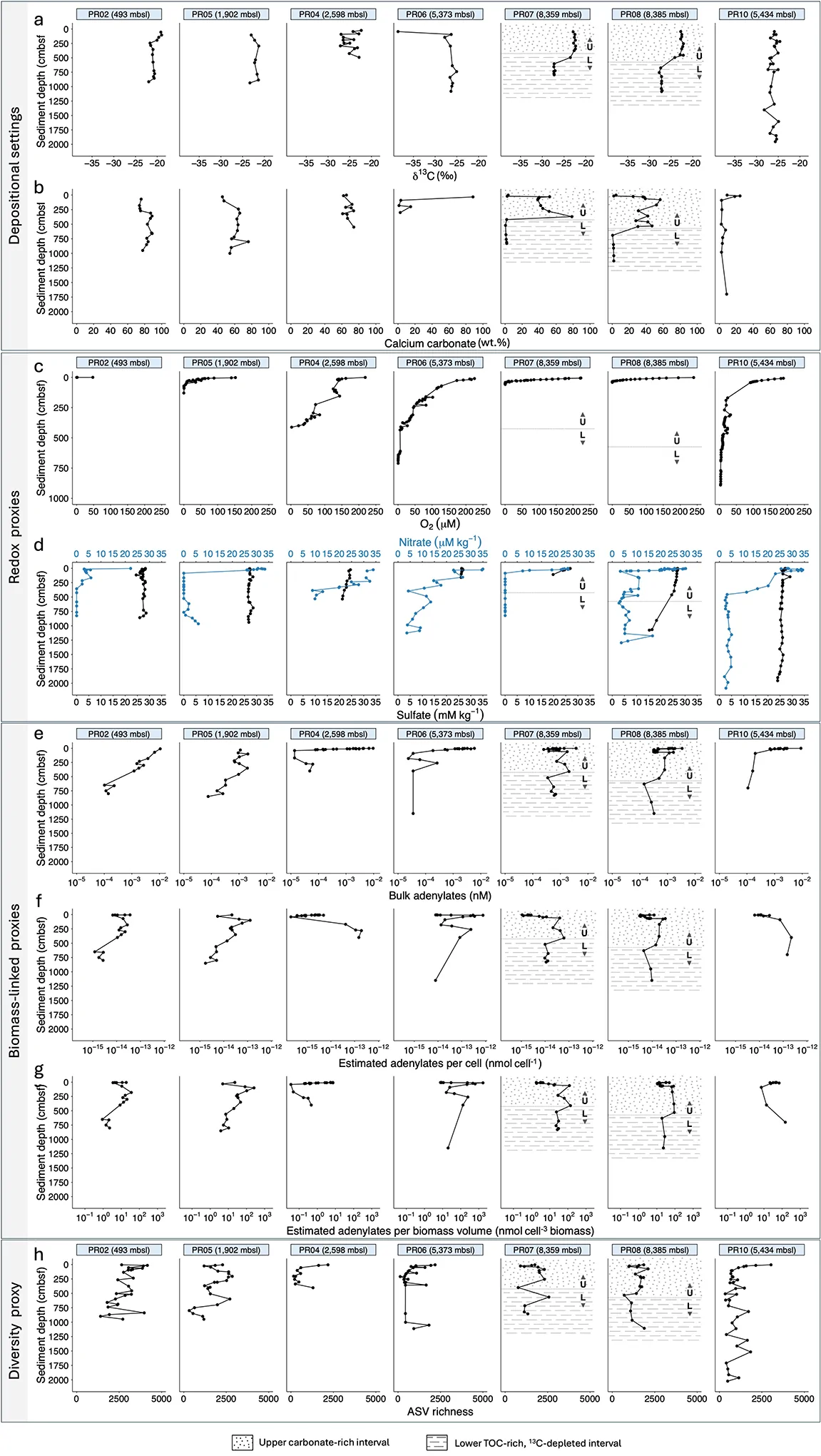
Geochemical, redox, biomass-linked and diversity profiles across the Puerto Rico Trench transect. **a,b,** δ^13^C_org_ (‰) and calcium carbonate (CaCO_3_; wt.%) profiles. **c,d,** Oxygen (μM), nitrate (μM kg^−1^) and sulfate (mM kg^−1^) profiles. **e–g,** Total adenylate (ATP + ADP + AMP; nM) and corresponding values normalized to cell abundance and cellular biovolume (nmol cell^−1^; nmol cm^−3^ biomass), respectively. **h,** Rarefied amplicon sequence variant (ASV) richness. Dashed lines mark the boundary between the upper carbonate-rich (U) and lower TOC-rich, ^13^C-depleted (L) intervals at PR07 and PR08.

**Extended Data Fig. 2 |:**
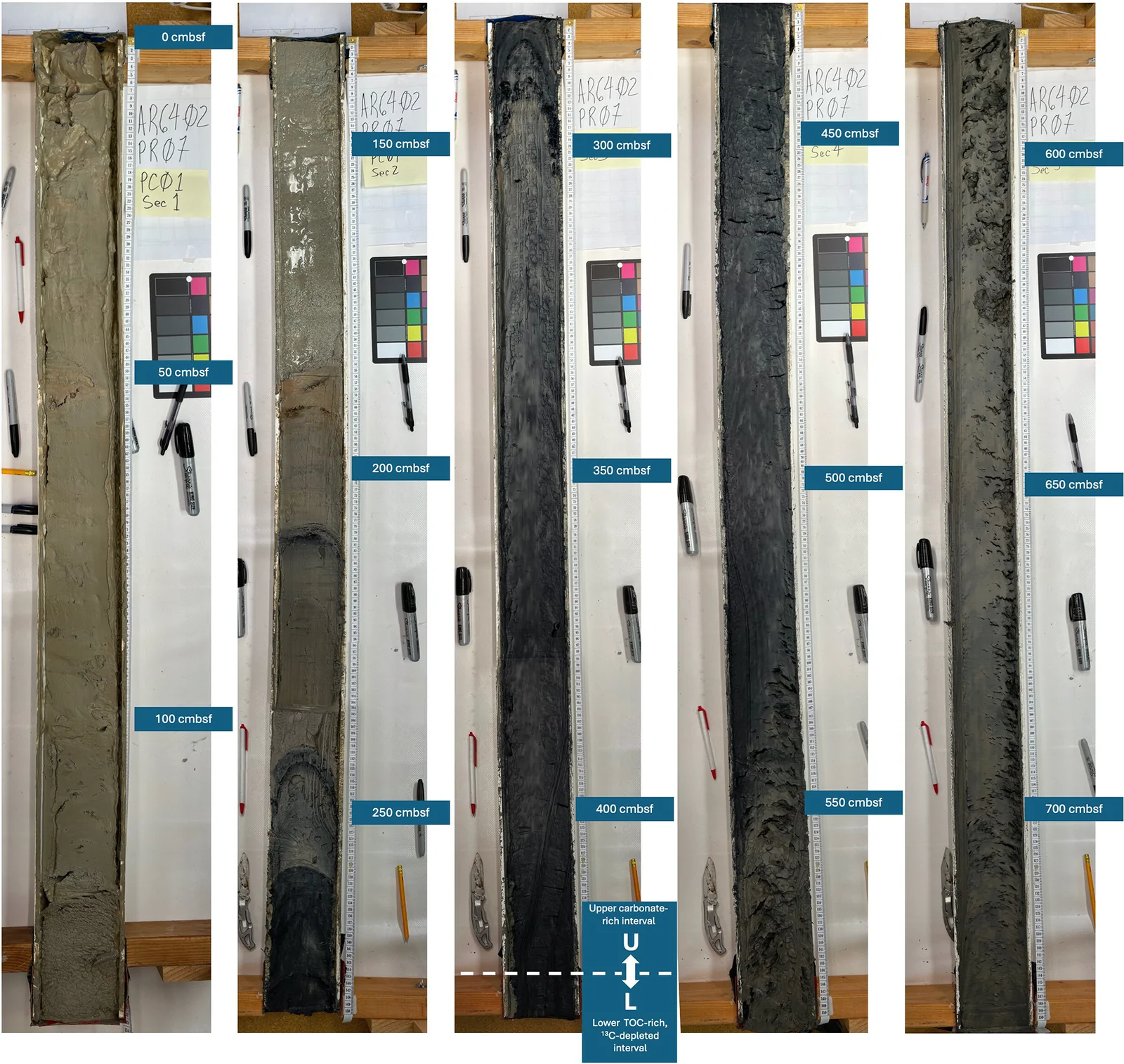
Lithological transition in PR07 trench-axis sediments. Split-core images showing the transition from the lighter-colored upper carbonate-rich interval (U) to the darker-colored lower TOC-rich, ^13^C-depleted interval (L). The boundary between intervals occurs at approximately 425 cmbsf.

**Extended Data Fig. 3 |:**
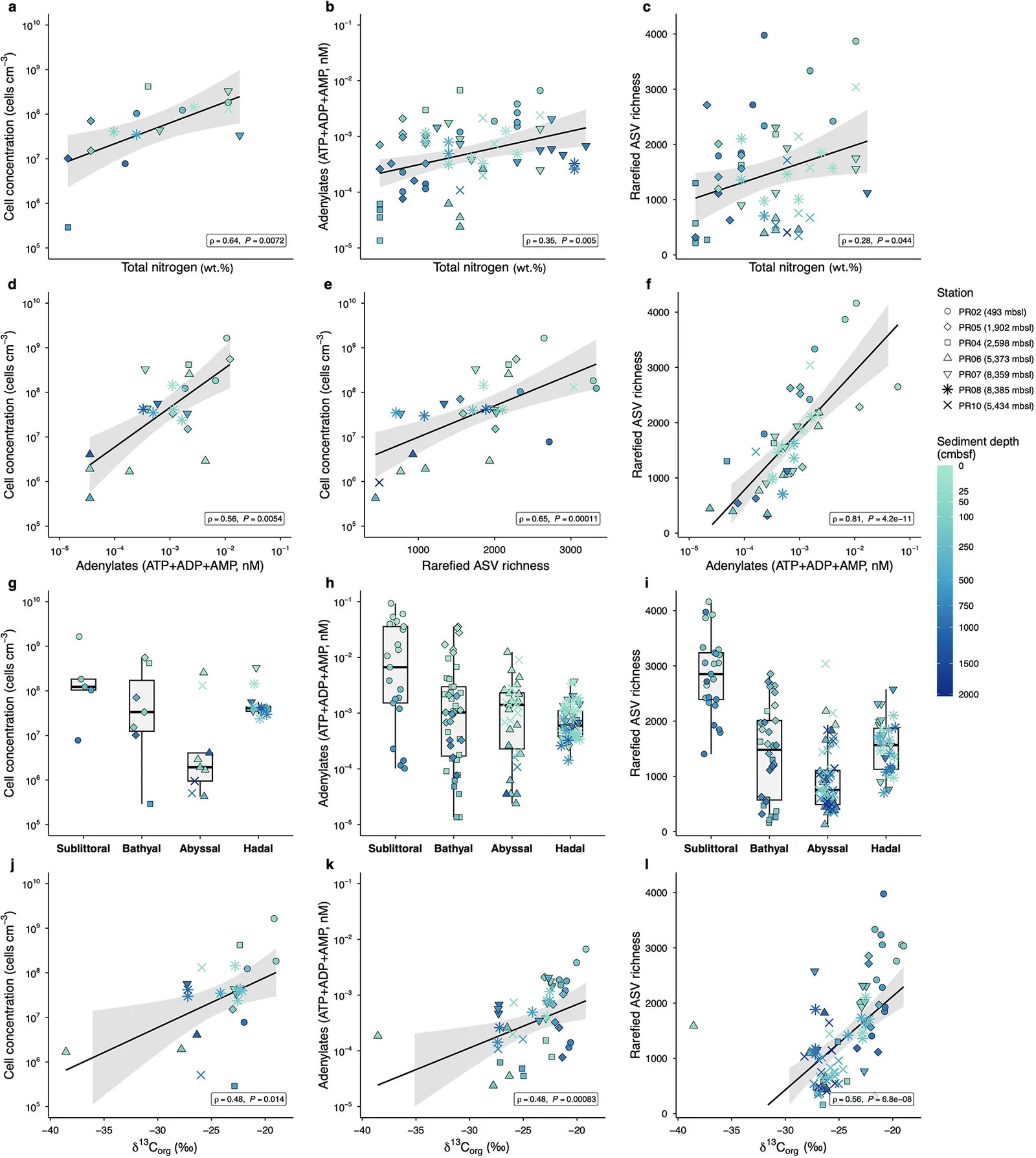
Relationships among organic-matter properties and microbial proxies across the Puerto Rico Trench transect. **a–c,** Relationships of total nitrogen content (TN; wt.%) with cell concentration (cells cm^−3^), total adenylate (ATP + ADP + AMP; nM) and rarefied amplicon sequence variant (ASV) richness, respectively. **d–f,** Pairwise relationships among these microbial proxies. **g–i,** Distributions of each proxy across depositional settings. **j–l,** Relationships of δ^13^C_org_ (‰) with cell concentration, total adenylate and rarefied ASV richness, respectively. Black lines and shading show fitted relationships and 95% confidence intervals. Insets report Spearman ρ and *P* values.

**Extended Data Fig. 4 |:**
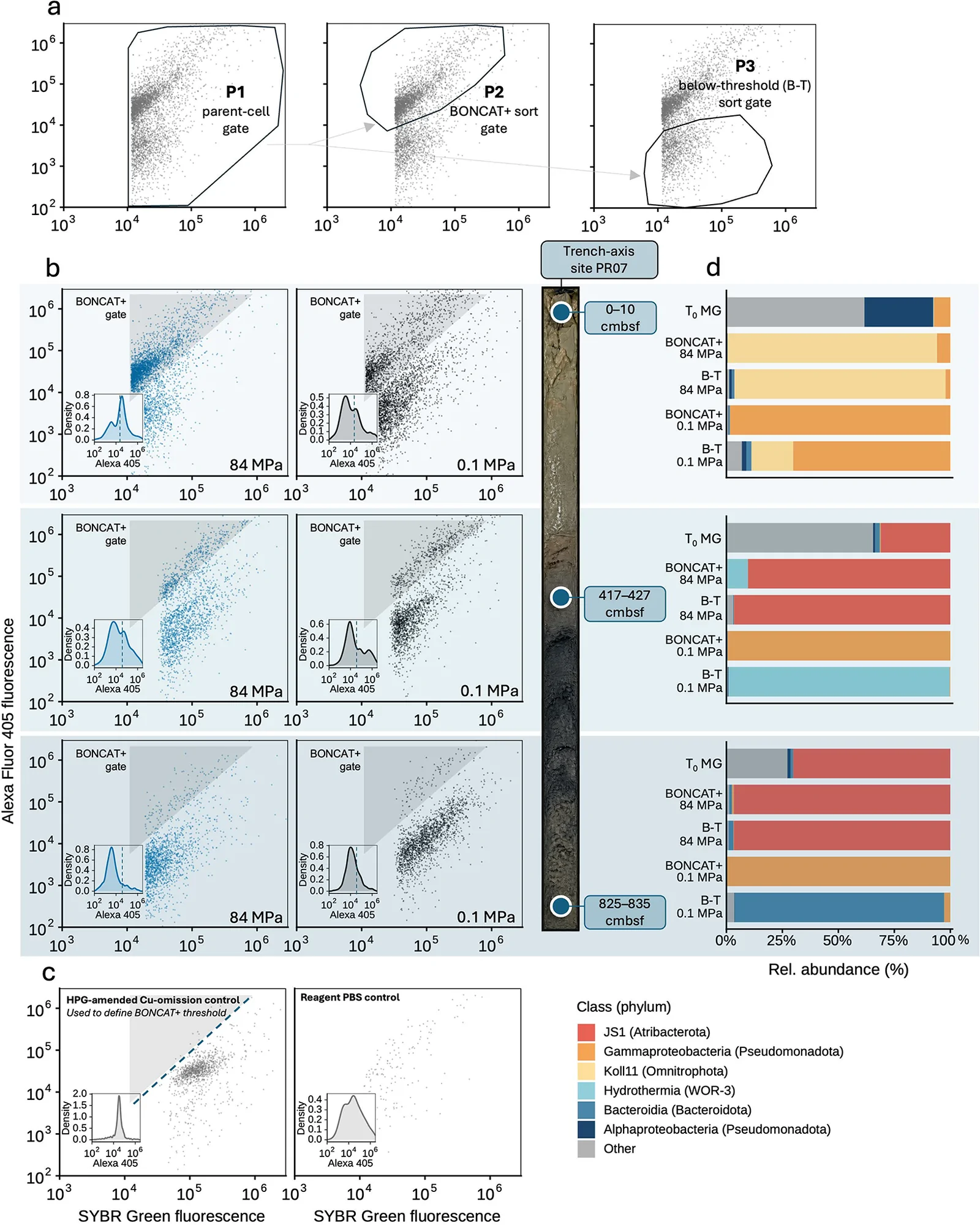
BONCAT-FACS gating and taxonomic composition of sorted PR07 sediment communities. **a,** Sequential FACS gating of parent cells (P1), BONCAT-positive cells (P2) and below-threshold (B-T) cells (P3). **b,** SYBR Green and Alexa Fluor 405 fluorescence during sorting of PR07 sediments at 0–10, 417–427 and 825–835 cmbsf incubated under atmospheric and *in situ* pressure; shading indicates the BONCAT-positive gate, and insets show Alexa Fluor 405 distributions. **c,** HPG-amended copper-omission control used to define the BONCAT-positive fluorescence threshold and reagent-only PBS control. **d,** Class-level taxonomic composition of T_0_ metagenomes and BONCAT-positive and below-threshold sorted fractions across sediment depths and pressure treatments.

**Extended Data Fig. 5 |:**
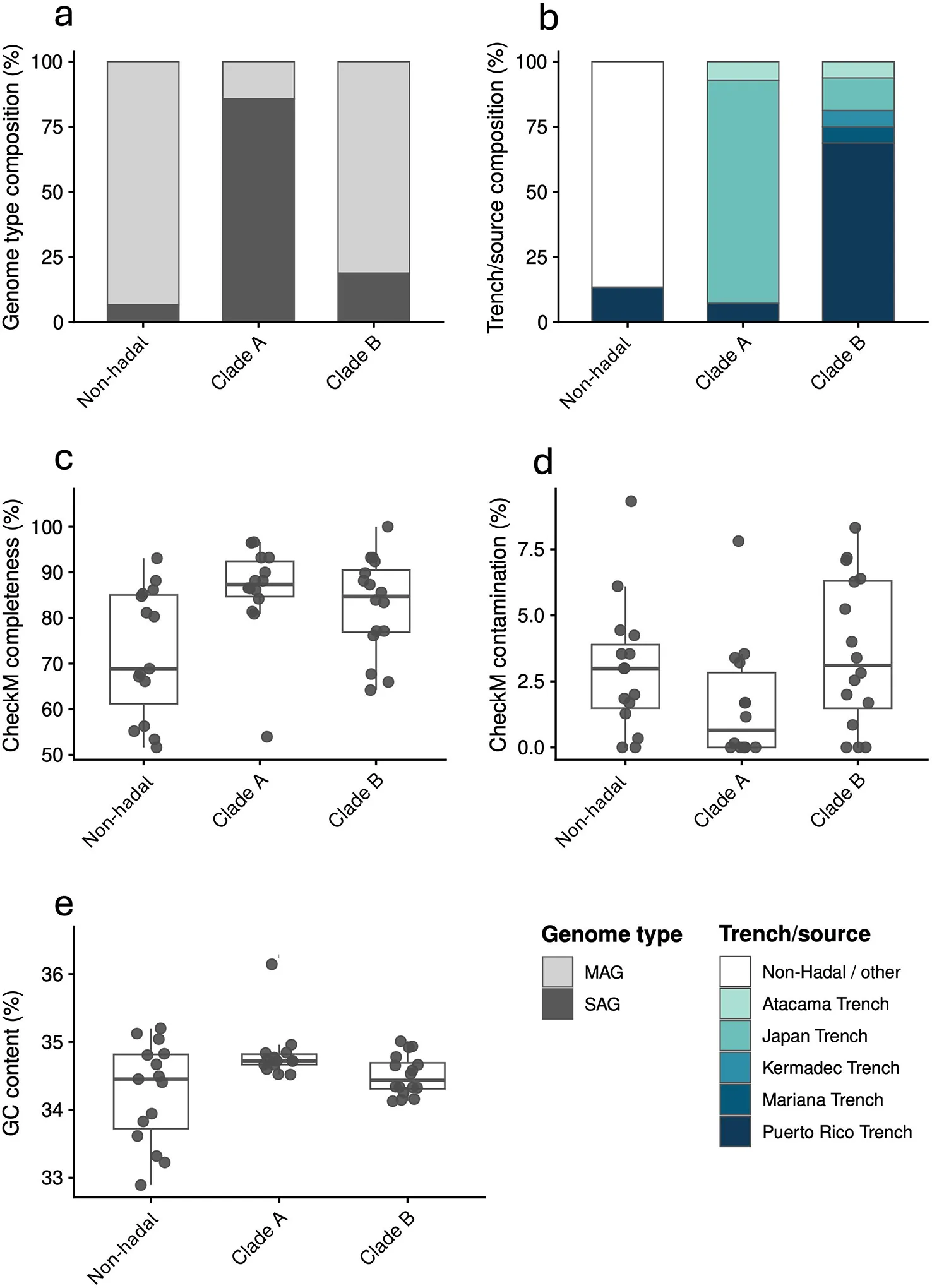
Composition and genome properties of the JS1 genome collection. a, MAG and SAG composition among non-hadal JS1 and hadal clades A and B. b, Trench and source composition of each genomic group. c–e, CheckM completeness (%), contamination (%) and GC content (%), respectively. Points represent individual genomes; boxes show medians and interquartile ranges.

**Extended Data Fig. 6 |:**
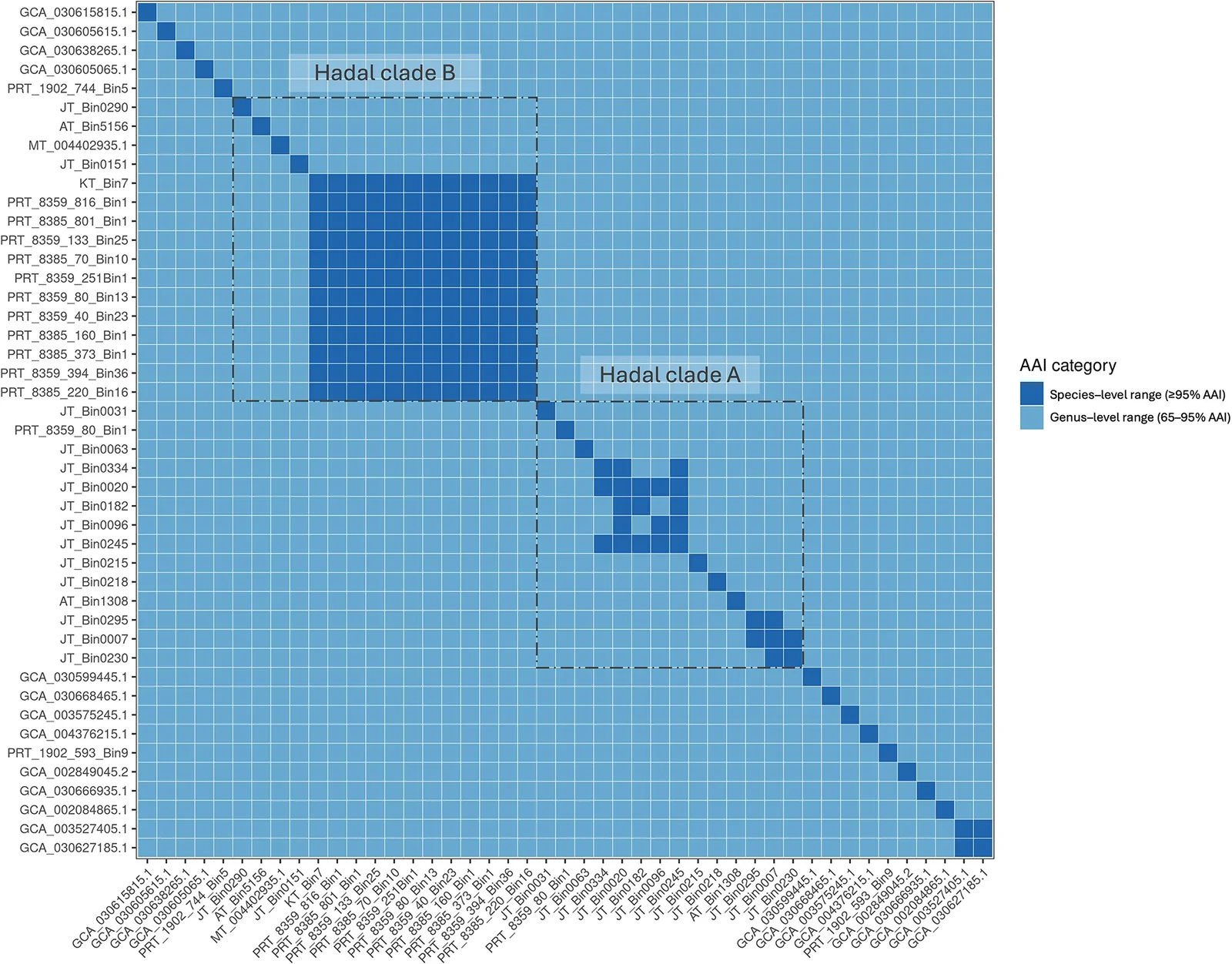
Pairwise average amino acid identity among JS1 genomes. Heatmap of pairwise average amino acid identity (AAI), with colors indicating genus-level (65–95% AAI) and species-level (≥95% AAI) ranges. Boxes delineate hadal clades A and B.

**Extended Data Fig. 7 |:**
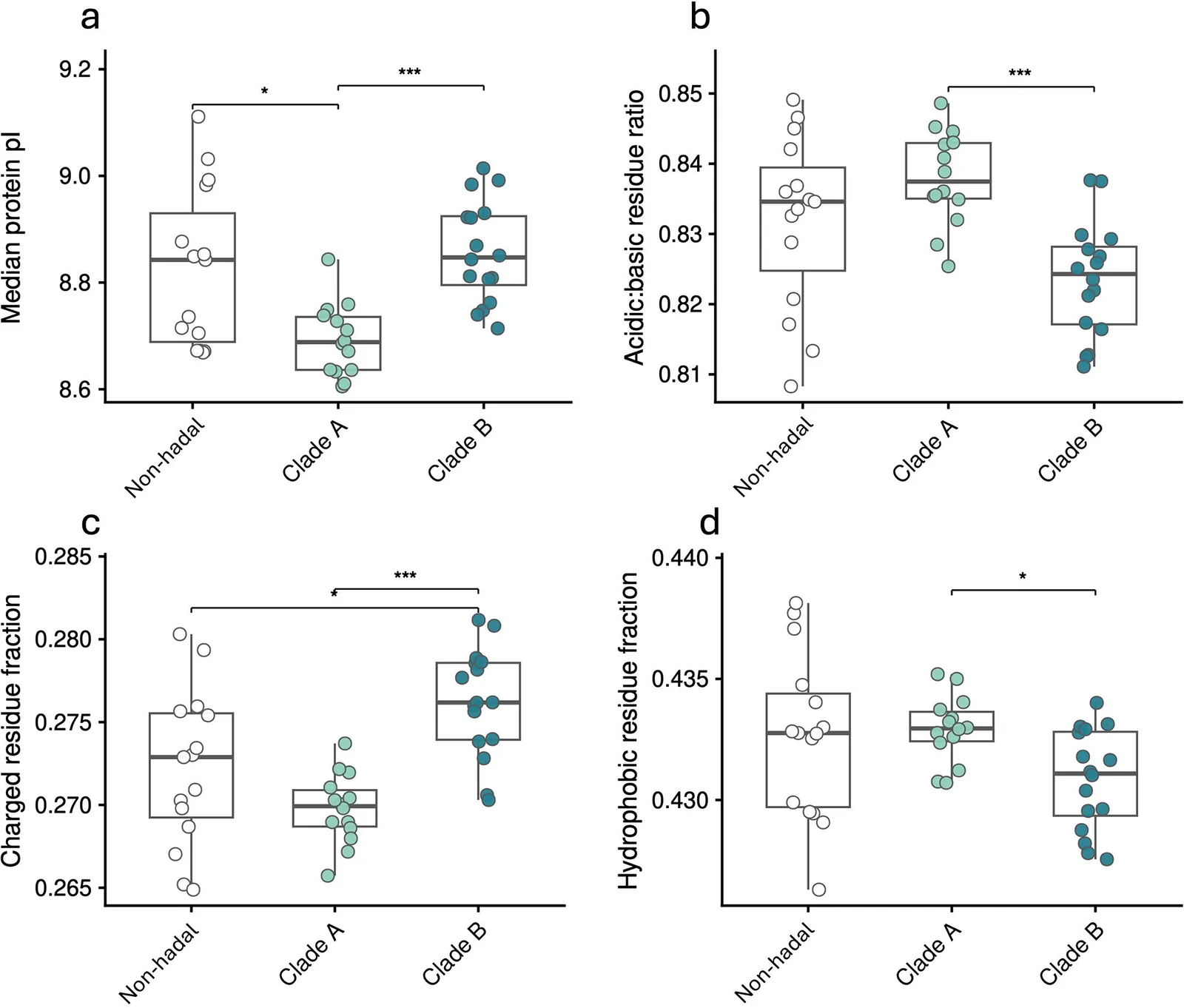
Proteome physicochemical properties across JS1 genomic groups. **a,** Median protein isoelectric point (pI). **b,** Acidic-to-basic residue ratio. **c,** Charged-residue fraction. **d,** Hydrophobic-residue fraction. Points represent individual genomes; boxes show medians and interquartile ranges. Pairwise Wilcoxon rank-sum tests with Benjamini–Hochberg-adjusted *P* values (*P < 0.05, **P < 0.01, ***P < 0.001).

## Supplementary Material

Supplement 1

## Figures and Tables

**Fig. 1 |:**
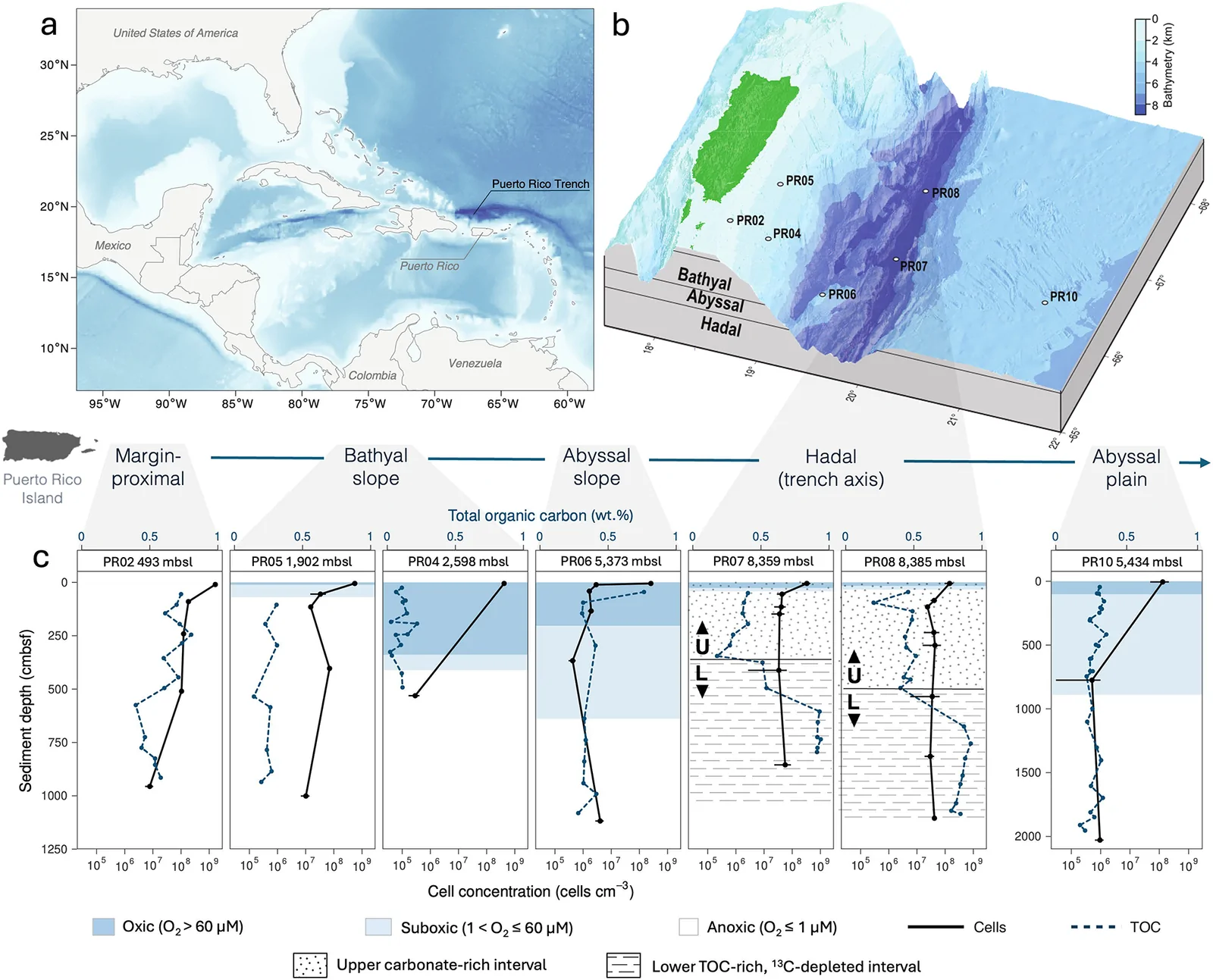
Organic matter, redox and microbial biomass vary across depositional settings, with pronounced heterogeneity at the trench axis. a, Regional location of the Puerto Rico Trench. b, Bathymetry and sampling sites. c, Sediment-depth profiles of cell concentration and total organic carbon (TOC; wt.%) across redox zones and depositional intervals.

**Fig. 2 |:**
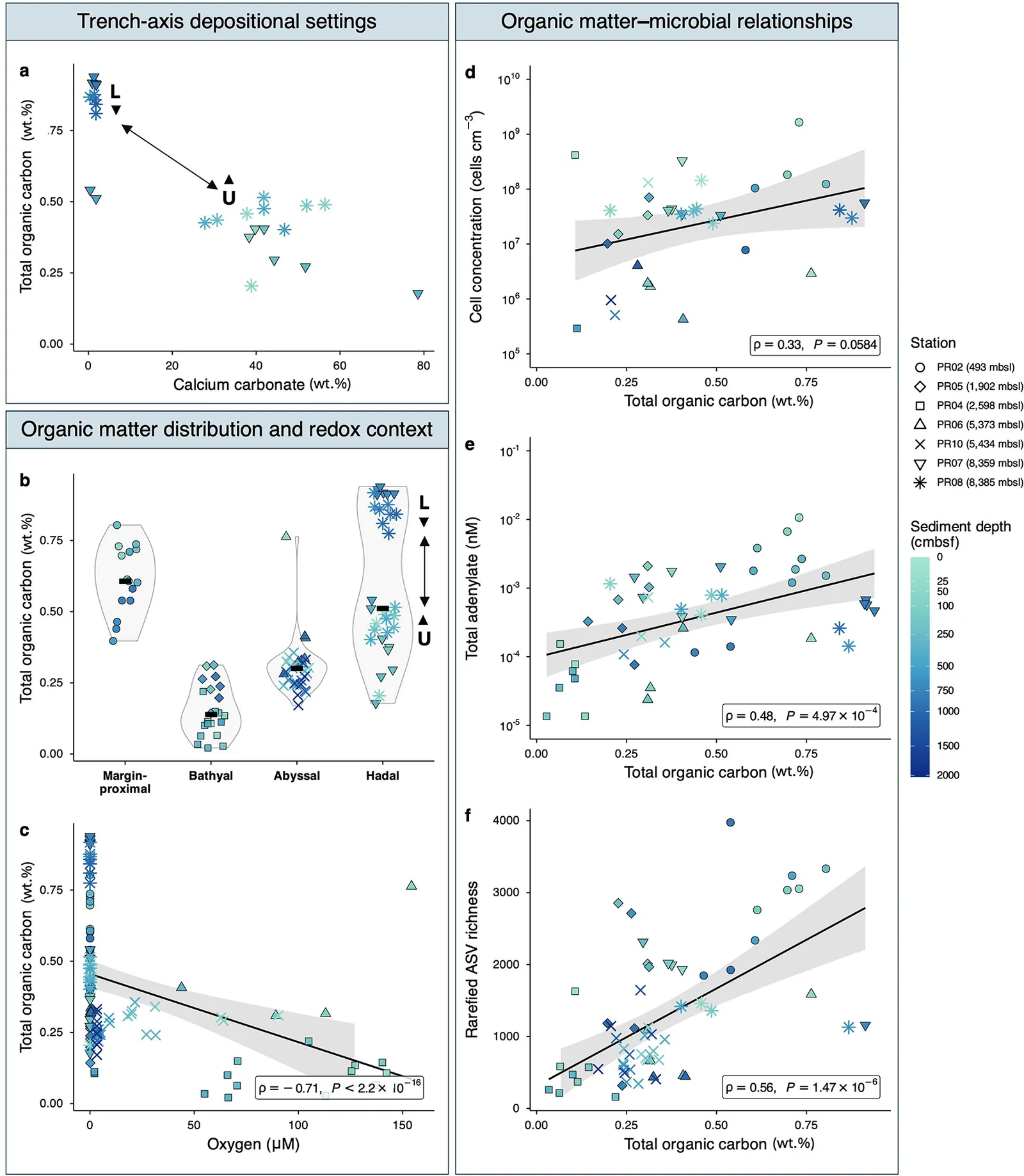
Organic matter covaries with microbial biomass and diversity across the transect but decouples in ^13^C-depleted trench-axis sediments. **a,** Relationship between total organic carbon (TOC; wt.%) and calcium carbonate (CaCO_3_; wt.%) in trench-axis sediments. **b,** TOC distributions across depositional settings. **c,** Relationship between TOC and oxygen concentration (μM). **d–f,** Relationships between TOC and cell concentration (cells cm^−3^), total adenylate (ATP + ADP + AMP; nM) and rarefied amplicon sequence variant (ASV) richness, respectively. Black lines and shading show fitted relationships and 95% confidence intervals; insets report Spearman ρ and *P* values.

**Fig. 3 |:**
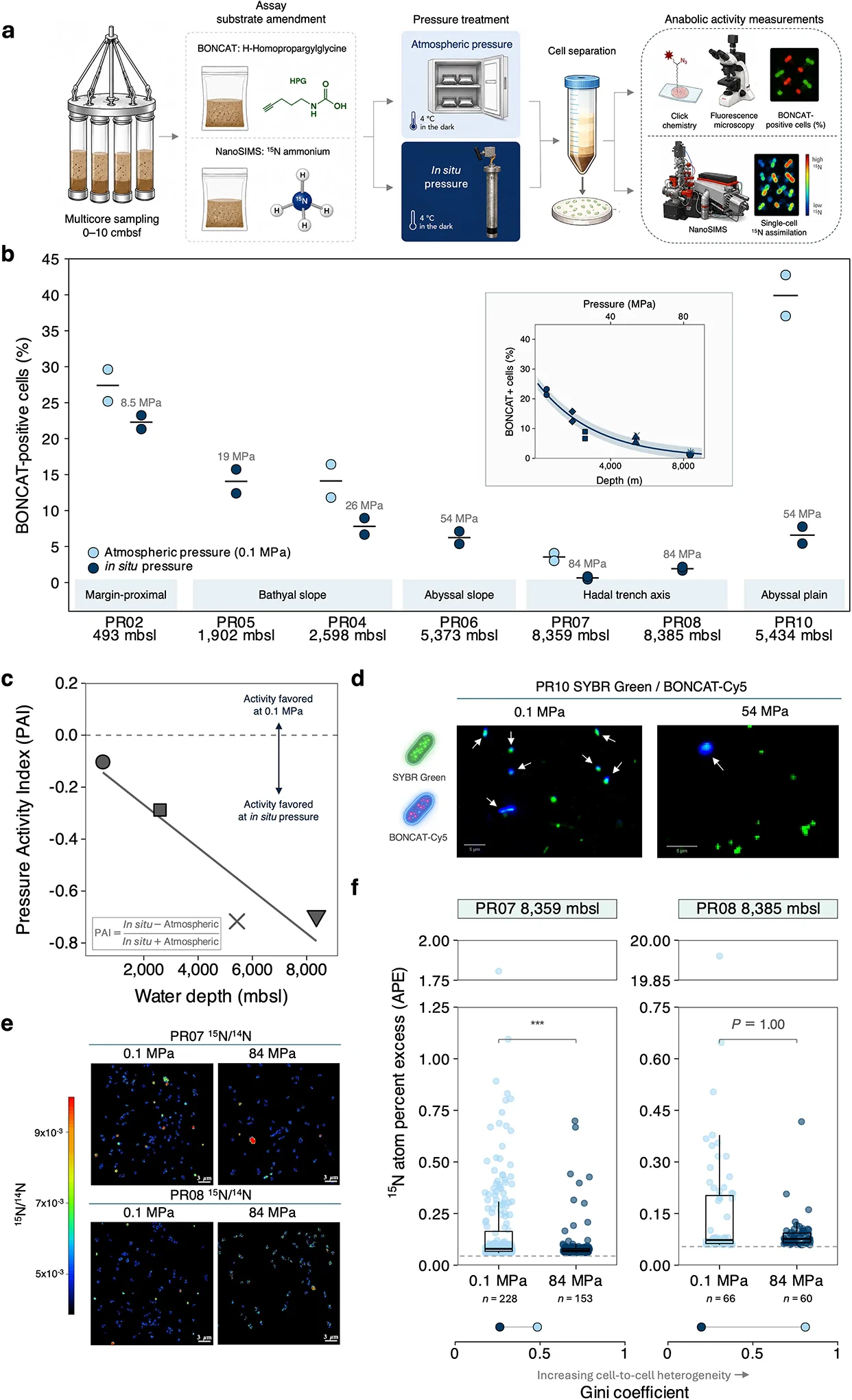
Atmospheric pressure increases the proportion of anabolically active cells in surficial sediments. **a,** Workflow for paired atmospheric and *in situ* pressure incubations followed by BONCAT and NanoSIMS. **b,** BONCAT-positive cells across the transect (two incubation replicates per sample and pressure treatment); inset shows the relationship between the *in situ*-pressure BONCAT-positive proportion and water depth. **c,** Pressure Activity Index (PAI), calculated from treatment means. **d,** Representative BONCAT fluorescence images from PR10; arrows indicate BONCAT-positive cells. **e**, Representative NanoSIMS ^15^N/^14^N images from PR07 and PR08; areas outside cellular regions of interest (ROIs) were set to zero and are shown in black. **f,** Cellular ^15^N atom percent excess (APE) among ^15^N-active cells from PR07 and PR08; dashed lines indicate the threshold used to classify ^15^N-active cells. Boxes show medians and interquartile ranges; *n* indicates cells analyzed. Two-sided Wilcoxon rank-sum tests with Bonferroni correction (***P < 0.001; PR08, adjusted *P* = 1.00).

**Fig. 4 |:**
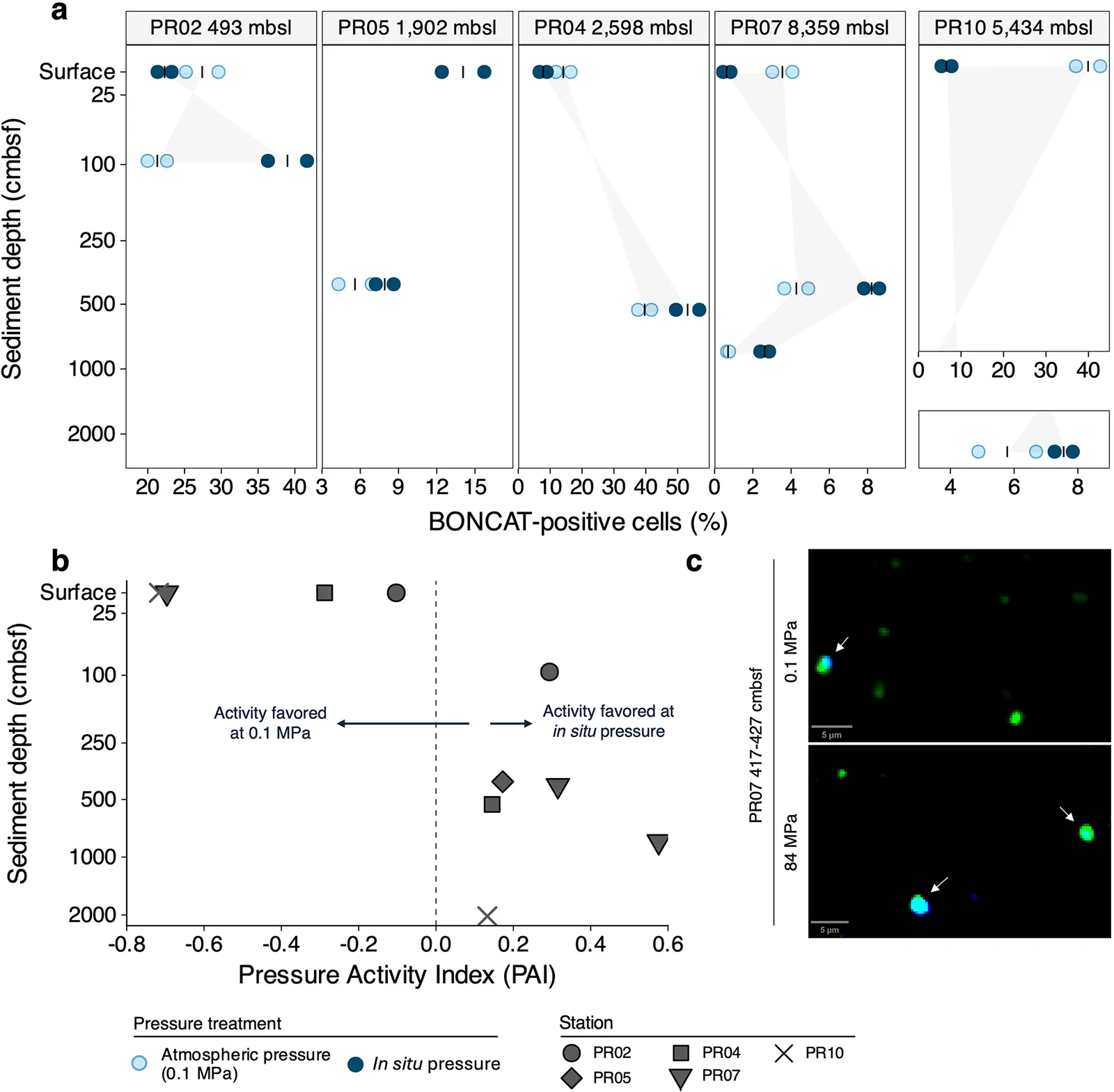
Microbial pressure responses reverse with burial from atmospheric-pressure-favored to *in situ*-pressure-favored activity. **a,** BONCAT-positive cells in surface and subsurface sediments under atmospheric and *in situ* pressure (two incubation replicates per sample and pressure treatment). **b,** Pressure Activity Index (PAI) across sediment depths, calculated from treatment means. **c,** Representative BONCAT fluorescence images from PR07 at 417–427 cmbsf under atmospheric and *in situ* pressure; arrows indicate BONCAT-positive cells.

**Fig. 5 |:**
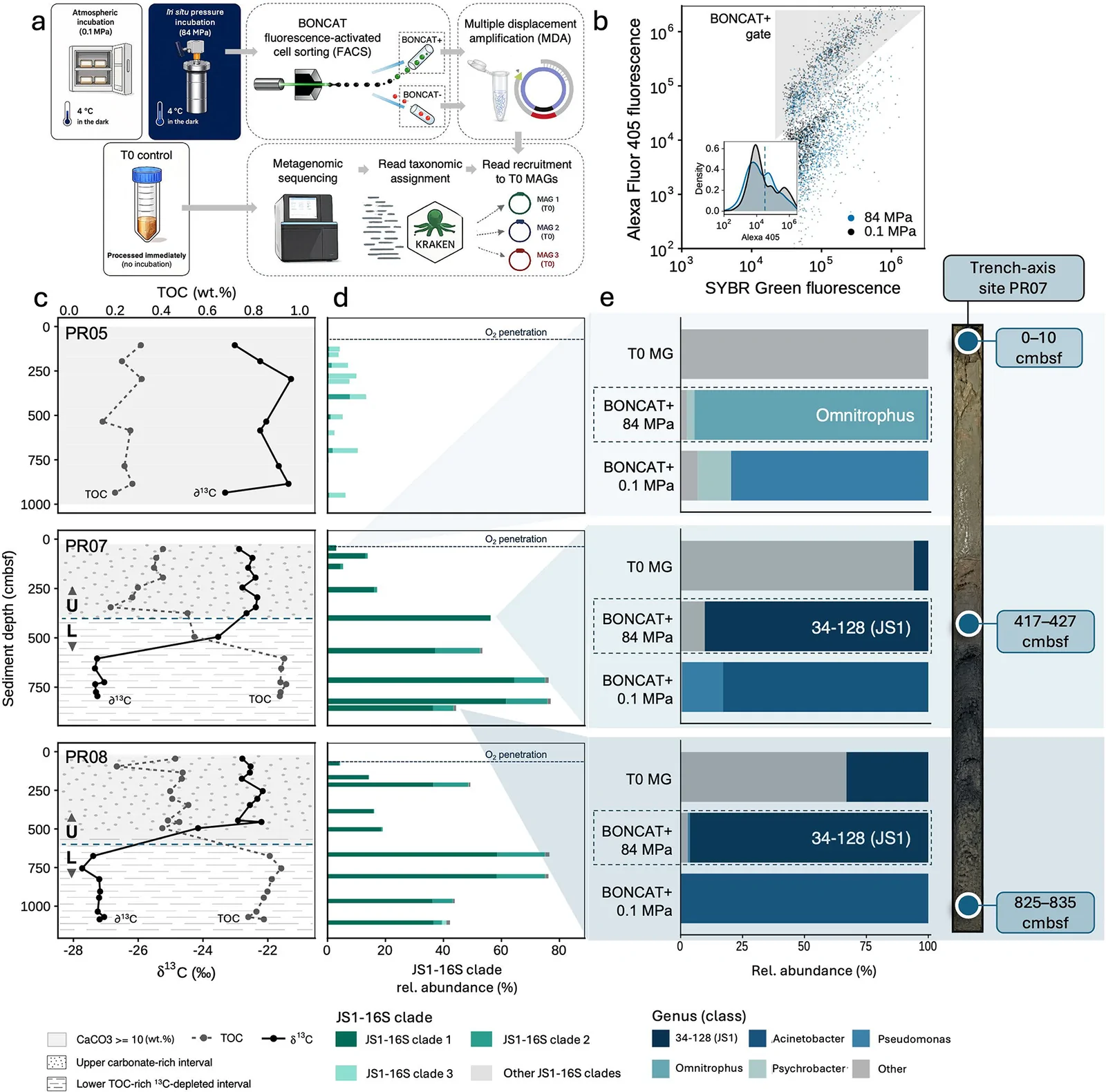
Abundant trench-axis JS1 populations are associated with distinct depositional intervals and anabolic activity under *in situ* pressure. **a,** BONCAT-FACS-seq workflow for atmospheric and *in situ* pressure incubations and T_0_ metagenomic controls. **b,** Representative BONCAT-positive FACS gate and Alexa Fluor 405 fluorescence distributions. **c,** Total organic carbon (TOC; wt.%) and δ^13^C_org_ (‰) profiles across depositional intervals at PR05, PR07 and PR08. **d,** Relative abundance of JS1 16S rRNA gene clades with sediment depth. **e,** Genus-level composition of T_0_ metagenomes and BONCAT-positive fractions under atmospheric and *in situ* pressure at trench-axis site PR07.

**Fig. 6 |:**
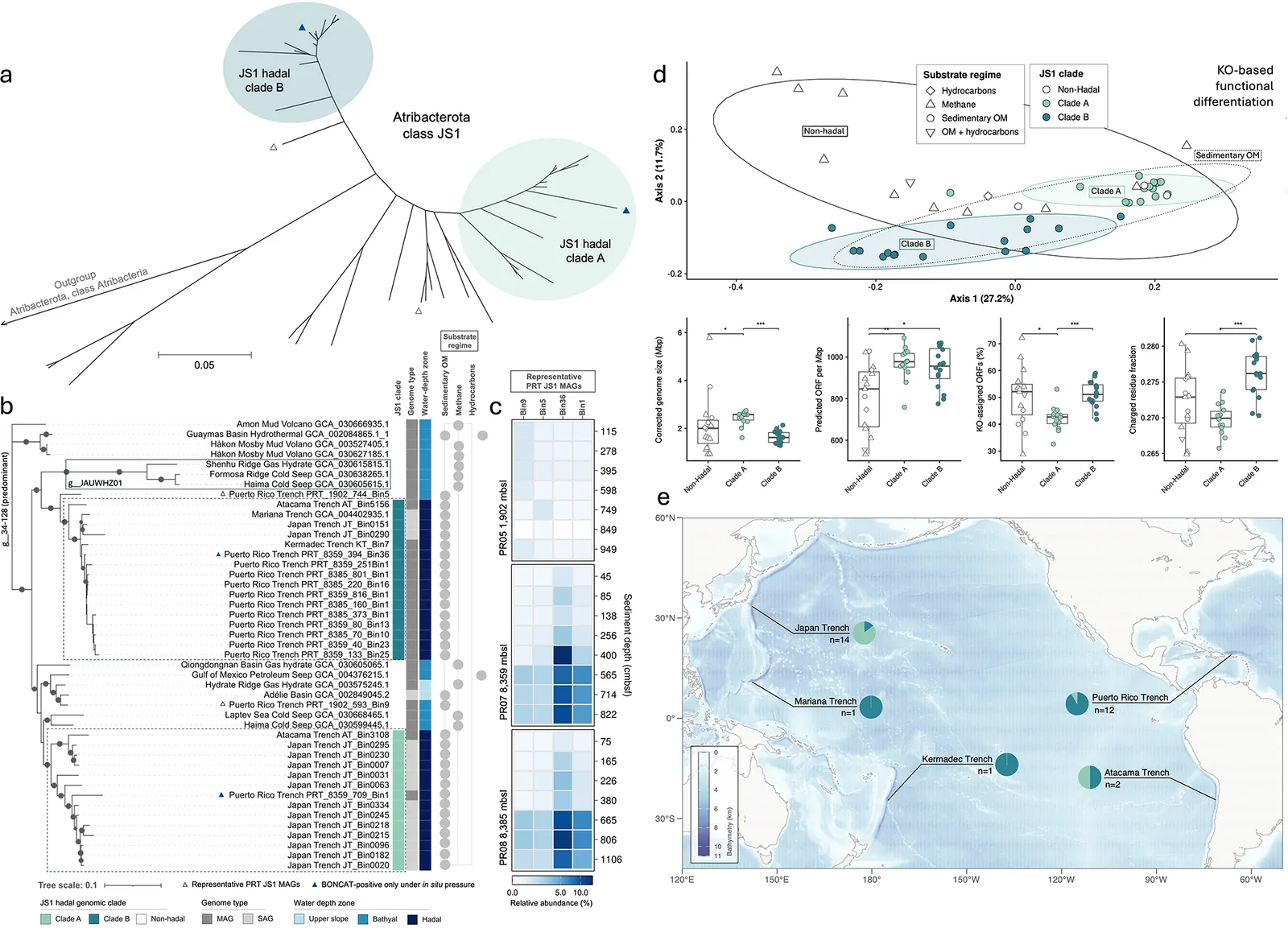
*In situ*-pressure-active JS1 populations belong to genomically differentiated clades widespread across hadal trenches. **a,** Simplified phylogenomic topology of JS1, highlighting hadal clades A and B. **b,** Detailed maximum-likelihood JS1 phylogeny annotated by genome type, water-depth zone and substrate regime. **c,** Relative abundance of representative Puerto Rico Trench (PRT) JS1 MAGs across sediment depths at PR05, PR07 and PR08. **d,** KEGG Ortholog (KO)-based functional differentiation and genome properties among non-hadal JS1 and hadal clades A and B. KO-profile differences were assessed by PERMANOVA; boxes show medians and interquartile ranges, with asterisks indicating pairwise Wilcoxon rank-sum tests with Benjamini–Hochberg-adjusted *P* values (*P < 0.05, **P < 0.01, ***P < 0.001). **e,** Geographic distribution and number of publicly available JS1 MAGs assigned to hadal clades A and B across five hadal trenches.

## Data Availability

The sequencing data generated in this study, together with the Puerto Rico Trench sequence dataset analyzed here, will be deposited in the NCBI Sequence Read Archive and made publicly available upon submission of the manuscript to a peer-reviewed journal.

## Code availability

Custom code used for bioinformatic and statistical analyses in this study will be made publicly available upon submission of the manuscript to a peer-reviewed journal.
